# Determination of the Value of the Faraday With a Silver-Perchloric Acid Coulometer

**DOI:** 10.6028/jres.064A.040

**Published:** 1960-10-01

**Authors:** D. Norman Craig, James I. Hoffman, Catherine A. Law, Walter J. Hamer

## Abstract

An accurate value of the faraday has been determined by the electrolytic dissolution of metallic silver in aqueous solutions of perchloric acid. Standards of electric current, mass, and time as maintained by the National Bureau of Standards were utilized in the determinations. The electric current was measured in terms of the standards of electromotive force and electrical resistance. Silver of high purity, freed from oxygen, was used.

The value of the faraday was found to be

$$\begin{array}{l} \text{faraday}=96516.5\pm 2.4\;\text{coulombs}\;\text{gram-equivalen}t^{-1}\left(\text{physical}\;\text{scale}\right),\\ \text{faraday}=96490.0\pm 2.4\;\text{coulombs}\;\text{gram-equivalen}t^{-1}\left(\text{chemical}\;\text{scale}\right). \end{array}$$

These values were obtained using 107.9028±0.0013 and 107.8731±0.0013, for the atomic weight of silver on the physical and chemical scales, respectively.

The electrochemical equivalent of silver was found to be

$$\text{electrochemical equivalent of silver}=1.117972\pm 0.000019\;\text{milligram coulom}b^{-1}.$$

This value may be used in an alternate method of defining the ampere in absolute value, namely, that steady current which will dissolve 1.117972 milligrams of silver per second and depends only on the standards of mass and time. The indicated uncertainties are overall limits of error based on 95 percent confidence limits for the mean and allowances for the effects of known sources of possible systematic error.

## 1. Introduction

The classic method for the determination of the faraday involves the electrolytic deposition of silver on platinum from an aqueous solution of silver nitrate. This method has been extensively studied and under closely specified conditions [1]^1^ was used for many years in defining the international ampere. In the original work at the National Bureau of Standards the national standards of electric current, mass, and time were used, and great emphasis was placed on the reproducibility of the method as a check on the stability of the existing standards of electromotive force (Weston cell) and electrical resistance (mercury ohm). In 1916 Rosa and Vinal [2] summarized the work that had been done with the silver coulometer not only in yielding a value of the faraday but also in attaining conditions wherein reproducible results could be obtained. At that time the consensus was that the silver coulometer gave highly accurate as well as reproducible results and yielded a value of the faraday accurate to within 0.001 percent (10 ppm).

However, 4 years earlier (in 1912) Washburn and Bates [3] had proposed an improved^2^ iodine coulometer for which high reproducibility and accuracy were claimed. In 1914 Vinal and Bates [8, 9] made a direct comparison of the silver and iodine coulometers in the laboratories of NBS and found that the two methods yielded different values for the faraday. Since the two types of coulometers were run in series the ratio of the results given by the two methods was independent of the electrical units and the durations of the runs. The result obtained with the iodine coulometer was 220 ppm higher than that given by the classic silver method. To date this discrepancy has not been resolved conclusively.^3^

In the ensuing years the silver coulometer has been criticized on two main counts: (1) a partial separation of the isotopes of silver may occur during the deposition, and (2) the silver deposits may contain occlusions of electrolyte (silver nitrate), acid (nitric acid), and water, alone or in combination. The first criticism does not appear to be valid judging from the results of the most precise determinations [12, 13] on the atomic weight of silver wherein in some cases the silver was reported to have been purified by repeated electrolysis. It will be shown later in this paper that no measurable separation of the isotopes of silver occurs on one, two, or three successive electrolyses; therefore, the first criticism is, in fact, not pertinent. Vinal and Bovard [14] studied the occlusion problem at length and observed a loss in weight when the silver deposits were heated to slightly above 600° C. They attributed this loss in weight to occluded foreign matter which was released on heating. This loss in weight amounted to a correction of only 0.004 percent in the faraday and was insufficient to explain the discrepancy between the silver and iodine values. Others [15 to 18] who worked on the problem found occlusions, the amounts of which varied from 0.004 to 0.016 percent. In all this work it is evident that corrections for occlusions remain uncertain.

Although the iodine coulometer has received less criticism in the ensuing years, the iodine value for the faraday has not been generally accepted. Vinal and Bates discussed the possible sources of error in each method but did not indicate a preference for either method. Instead they recommended that a rounded value of the average of the values given by the two methods, namely 96,500 international coulombs per gram-equivalent, be adopted for general use. It is somewhat surprising that this situation has persisted more or less to the present time. One reason for this lies in the fact that the national standardizing laboratories were engaged in work leading to the conversion from international to absolute electrical units. Two World Wars had caused delays in the necessary experimental work. When this conversion was finally realized on January 1, 1948, it was then deemed an appropriate time to redetermine the faraday. Several electrochemical methods were considered and of all those considered the one presented here was considered to be the most promising.

Because of the two main criticisms of the silver coulometer a method wherein these would not be valid would be desirable. Electrolytic dissolution (or corrosion) of metallic silver at once suggests itself. Occlusions could play no part in electrolytic dissolution of pure silver. Also, isotopic separation would be eliminated a priori (see p. 393), since whole (or bulk) silver would be uniformly dissolved electrolytically. This method using an electrolyte of silver nitrate was used in a few qualitative experiments by Mascart [19] and Gray [20]. Also, Rosa, Vinal, and McDaniel [21] investigated the electrolytic dissolution of silver in silver nitrate in studies of the reversibility of the silver coulometer. Their results, although generally overlooked, indicate good reversibility. Their anode consisted of silver deposited electrolytically on platinum and the loss in weight agreed well indeed with the gain in weight in a conventional coulometer, run in series, wherein the silver was deposited electrolytically. In two runs, electrolytic deposition gave 96,494 whereas electrolytic dissolution of silver gave 96,488 international coulombs gram-equivalent^−1^.

These two experiments of Rosa, Vinal, and McDaniel, therefore, showed that the silver coulometer was reversible to at least 6 parts in 100,000. However, the silver anodes that were corroded had been prepared by electrolytic deposition and were, therefore, subject to occlusion errors. Furthermore, Rosa, Vinal, and McDaniel stated that the amount of silver on the platinum bowl should not fall below a critical amount and that a large surface of silver should be used. If the current density became too high, or the amount of silver on the platinum too small, current flowed from the underlying platinum whereby simultaneous reactions occurred at the anode. Although not stated by Rosa, Vinal, and McDaniel, silver oxide or oxygen probably formed at the exposed platinum in the neutral silver nitrate solution if the current density became too high. Since silver oxide is not appreciably soluble in silver nitrate it would be included in the anode sediment (the silver that fell from the anode during the electrolysis) which must be weighed at the end of an experiment; this silver oxide would thus cause errors. Likewise, if oxygen were formed at the anode in neutral silver nitrate solution it would escape to the atmosphere also causing errors in faraday determinations.

It is obvious, therefore, if a method wherein silver is electrolytically dissolved is to be considered seriously for a determination of the faraday, these sources of error must be eliminated or reduced to a minimum. This may be done (1) by using silver anodes thereby eliminating platinum entirely, and (2) by using an acid electrolyte in which silver is highly stable but in which the silver salt is freely soluble. By using an acid solution the formation of silver oxide would be precluded as well as the formation of oxygen (see below). The problem then rested in finding the proper electrolyte to use with silver anodes so that the electrochemical reaction:

$$\text{Ag}\;\left(\text{metal}\right)\to {\text{Ag}}^{+}\left(\text{ions}\right)+\epsilon \left(\text{electrons}\right)$$

would proceed quantitatively without any side reactions.

Of all the electrolytes considered or studied it was found that perchloric acid was the most suitable and was used in some preliminary studies by Craig and Hoffman [22]. The stability (or solubility) of silver in perchloric acid, in the absence of current flow, has recently been studied in detail [23]. It was found that silver is highly stable in perchloric acid (20 wt %), more stable in perchloric acid (20 wt %) containing a small amount (0.5 wt %) of silver Perchlorate, and practically inert in conductivity water. The stability is such as to cause negligible errors in faraday determinations.

Although occlusion errors are eliminated in the method described herein, the purity of the silver remains a most important problem. The purity of deposits (old method) and the purity of silver used as anodes (new method) are equally important. Fortunately, however, extremely pure silver may be prepared for use in the present method whereas the determinations of the occlusions in silver deposits is extremely difficult. The most insidious impurity that is encountered in preparing pure silver is oxygen either as the element or combined as silver oxide. When it is present as free or uncombined oxygen it introduces an error which is proportional to its concentration. When combined as the oxide 16 parts of oxygen are equivalent to 232 parts of silver oxide which is chemically soluble in perchloric acid, and, accordingly, its presence as combined oxygen in the starting silver would cause appreciably larger errors in determining the faraday (this is discussed in more detail in appendix 1 to this paper).

## 2. Method and Coulometer

### 2.1. Method

The method consists simply of the electrolytic dissolution of metallic silver in aqueous solutions of perchloric acid (20 wt %) initially containing a small amount (0.5 wt %) of silver Perchlorate according to the electrochemical reaction:

$$\text{Ag}\;\left(\text{metallic}\right)\overset{{\text{HClO}}_{4}\;{\text{and AgClO}}_{4}}{\to }{\text{Ag}}^{+}\left(\text{ions}\right)+\epsilon^{-}\left(\text{electrons}\right)$$

wherein silver (the anode) is electrolytically oxidized to argentous ions. Metallic silver, in sheet or rod form, is used. It is weighed before and after the passage of a known current through the coulometer for a known time. Platinum gauze is used as the cathode. Silver Perchlorate is added to the perchloric acid in the small amount stated above to reduce the chemical solubility of silver in perchloric acid to an insignificant amount [23]. Sufficient silver oxide is initially added to the solution around the platinum cathode to provide silver ions so that silver is deposited throughout the run on the cathode and discharge of hydrogen ions and formation of hydrogen gas on the cathode are prevented; turbulence at the cathode and within the coulometer is thereby prevented.

### 2.2. Coulometer

**Figure 1:**
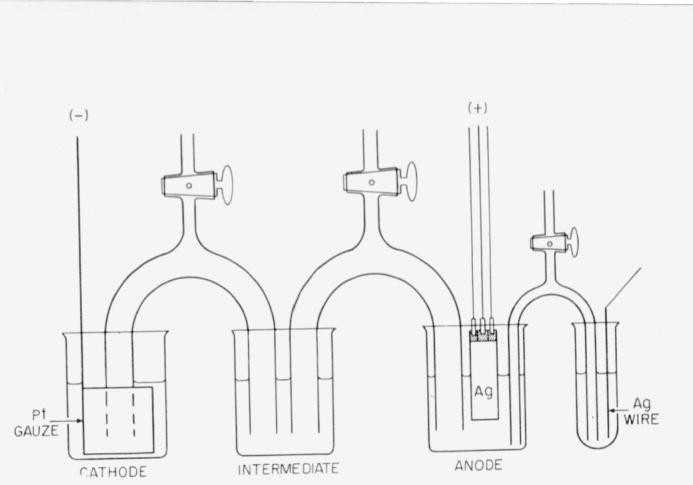
Schematic view of the coulometer.

**Figure 2:**
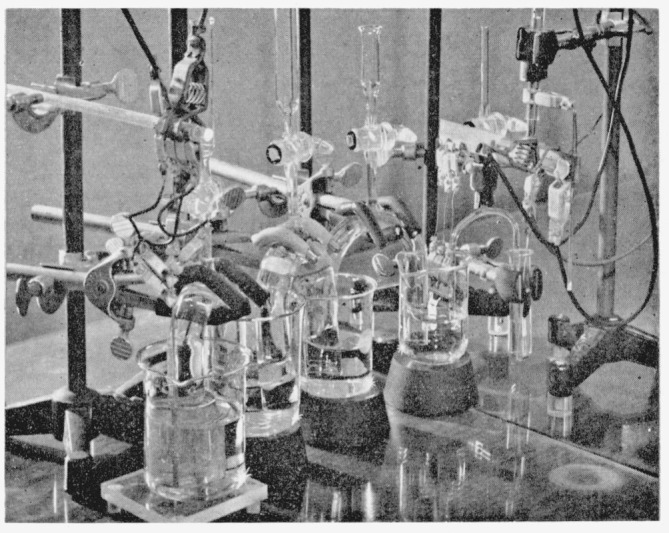
Photograph of coulometer with two intermediate beakers.

A schematic view of the coulometer is given in figure 1 and a photograph in figure 2. The silver anode is housed in a Pyrex beaker, the platinum cathode in a separate Pyrex beaker, and the solutions in the two beakers are brought into contact by two siphons and an intermediate beaker. In some cases two intermediate beakers and three siphons were used (see photograph). The coulometer was housed in a plastic cabinet to minimize exposure of the solution to dust and fumes. The diagram as shown in figure 1 is self-explanatory except for the reference silver electrode shown at the extreme right. The silver anode, during electrolysis, exhibits a potential, the value of which depends on the voltage drop in the solution, the concentration of silver ions in its vicinity, and on electrode polarization. As silver goes into solution during the electrolysis these effects change in a systematic manner; evidence of side or undesirable electrode reactions would be indicated by abrupt changes in the potential of the silver anode with respect to the reference (non-corroding) silver electrode. No abrupt change in the potential of the silver anode was observed in any of the coulometric runs.

Thermodynamic data may be used to show that the reaction at a silver anode in aqueous solutions of perchloric acid is the postulated one. The following electrochemical reactions may conceivably occur at silver anodes in aqueous solutions of perchloric acid containing a small amount of silver Perchlorate (such solutions contain Ag^+^, ClO_4_^−^, OH^−^, and H^+^ ions):

(a) $\text{Ag}\;\left(\text{metallic}\right)\to {\text{Ag}}^{+}+\epsilon^{-},\;E{}^{\circ}=-0.799\;v$
(b) $\text{Ag}\;\left(\text{metallic}\right)+{{\text{ClO}}_{4}}^{-},\left(aq\right)\to {\text{AgClO}}_{4}\left(aq\right)+\epsilon^{-},\;E{}^{\circ}=-0.799\;v$
[(c) $\left[\text{Ag}\;\left(\text{metallic}\right)+{\text{OH}}^{-},\left(aq\right)\to \left(½\right){\text{Ag}}_{2}O+\left(½\right)H_{2}O\;\left(l\right)+\epsilon^{-}\right]$,
(d) $\left(½\right)H_{2}O\;\left(l\right)\to \left(¼\right)O_{2}\left(g\right)+H^{+}+\epsilon^{-},\;E{}^{\circ}=-1.229\;v$
(e) $H_{2}O\;\left(l\right)\to \left(½\right)H_{2}O_{2}\;\left(aq\right)+H^{+}+\epsilon^{-},\;E{}^{\circ}=-1.77\;v$
(f) $\left(½\right)\text{Ag}\;\left(\text{metallic}\right)\to \left(½\right){\text{Ag}}^{++}+\epsilon^{-},\;E{}^{\circ}=-1.39\;v$
  where *aq* represents aqueous solution, *l* liquid, and *g* gas. Since AgClO_4_ is completely dissociated into silver and Perchlorate ions, reaction (b) is equivalent to reaction (a). Since Ag_2_O dissolves or reacts readily with HClO_4_ according to the reaction:
(g) $\left(½\right){\text{Ag}}_{2}O+{\text{HClO}}_{4}\to {\text{AgClO}}_{4}+\left(½\right)H_{2}O$
  reaction (c) combined with (g) is equivalent to reaction (b) which in turn is equivalent to reaction (a). The standard electrode (or oxidation-reduction) potentials are listed above for the pertinent eletrochemical processes [24].

Since the reaction requiring the least free energy will occur in preference to those of higher free energy, reactions other than reaction (a) will occur only to a negligible extent unless the overpotential for silver dissolution is high, of the order of 0.5 to 1.2 v. Measurements of the potentials of the silver anodes relative to the reference silver electrode throughout the experiments showed that these potentials, when concentration differences and *IR* drop were considered, were very close to the standard silver potential; accordingly, then, reaction (a) takes place in the electrolytic dissolution of silver in aqueous perchloric acid and the other reactions are negligible. In the above the standard potentials were used. Actually, the potentials corresponding to the perchloric acid-silver Perchlorate solution should be used. However, the differences between the standard potentials and those corresponding to the solutions of perchloric acid-silver Perchlorate are so small as not to alter the above conclusion.

Since the above electrode potentials relate to reactions in solutions free of oxygen some considerations must be given to errors that might have arisen from the oxygen dissolved in the solutions during the faraday determinations. Two reactions are involved, viz:

$$2\text{Ag}+\left(1/2\right)O_{2}\to {\text{Ag}}_{2}O,$$

followed by

$$\left(g\right)\;{\text{Ag}}_{2}O+2{\text{HClO}}_{4}\to 2{\text{AgClO}}_{4}+H_{2}O.$$

Both of these reactions are thermodynamically spontaneous. The first reaction is rate controlling and has an extremely slow rate under the conditions prevailing in the faraday determinations. In a careful study the errors that could arise from these reactions were found to be extremely small and are given in a previous paper [23].

The dimensions of the coulometer used with the purified anodes were for the most part as follows:

- Anode beaker—5 cm in diameter and 8 cm in height, especially constructed with rounded bottom to facilitate removal of anode sediment; cathode and intermediate beakers 7 cm in diameter and 9 cm in height.
- Siphons—2.1 cm in bore, 2.3 cm in outside diameter, 5 cm between legs, 25 cm along center of bore.
- Silver anode (sheet form)—4 cm in width, 0.02 to 0.03 cm thick, 1 to 3.5 cm in solution at start of electrolysis.
- Silver anode (elliptical rod form)—1.0 to 1.2 cm, major axis; 0.4 to 0.6 cm, minor axis; 1 to 3 cm in solution at start of electrolysis.
- Cylindrical platinum cathode—5 cm in height and 4 cm in diameter; 0.1 cm thick gauze.
- Reference electrode vessel—glass tube 9 cm in height and 1.5 cm in inside diameter.
- Reference silver electrode—wire 10 cm in length and 0.2 cm in diameter.
- Siphon (to reference electrode)—0.3 cm in inside diameter, 0.5 cm in outside diameter, 7 cm between legs, 24 cm along center of bore.

**Figure 3:**
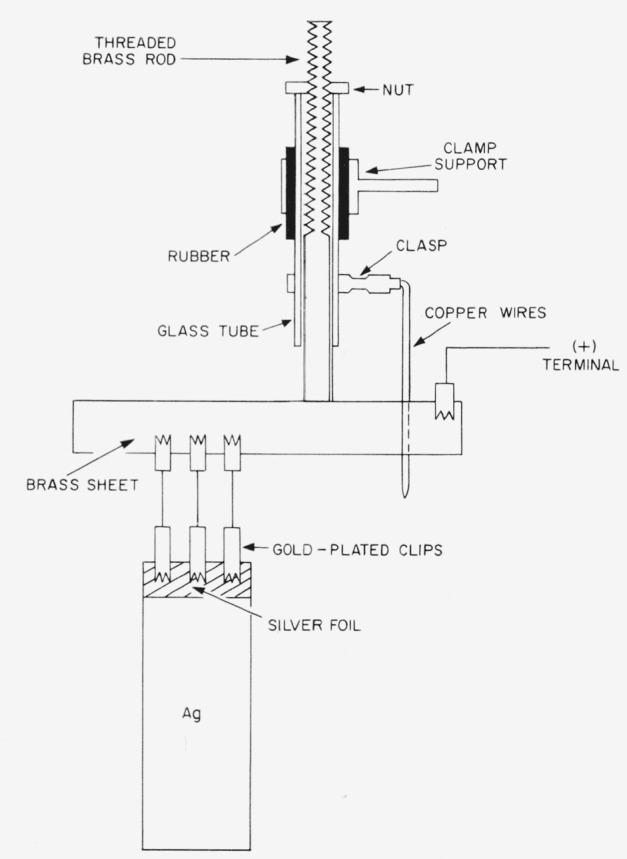
Schematic view of support for silver anode showing the method for lowering the anode into the coulometer solution.

During the electrolysis the silver anode dissolves electrolytically and means had to be provided to lower the anode at intervals into the solution. The means by which this was done for sheet anodes is shown in figure 3. The silver anode sheet was suspended in the solution by gold-plated clips. A piece of thin silver foil was bent over the entire top edge of the silver anode so that it was between the anode and the gold-plated clips. In all cases the silver foil and gold-plated clips were above the solution. These gold-plated clips were fastened to other clips by copper wires. The latter clips were clasped to a sheet of brass (⅛ in. thick) which was soldered at the upper middle edge to a brass rod, threaded at its upper end. The threaded end protruded through a glass tube and the location (height) of the sheet brass and, therefore, the silver anode was adjusted by a nut on the thread placed over the upper end of the glass tube. The glass tube was housed in a rubber stopper supported by a clamp on a ring stand. A clasp was placed around the glass tube and at the outer end of the clasp a copper wire was attached which looped around the sheet of brass; this wire prevented the silver anode from rotating during the lowering process. Current and potential leads were connected to the sheet of brass as indicated in figure 3.

**Figure 4:**
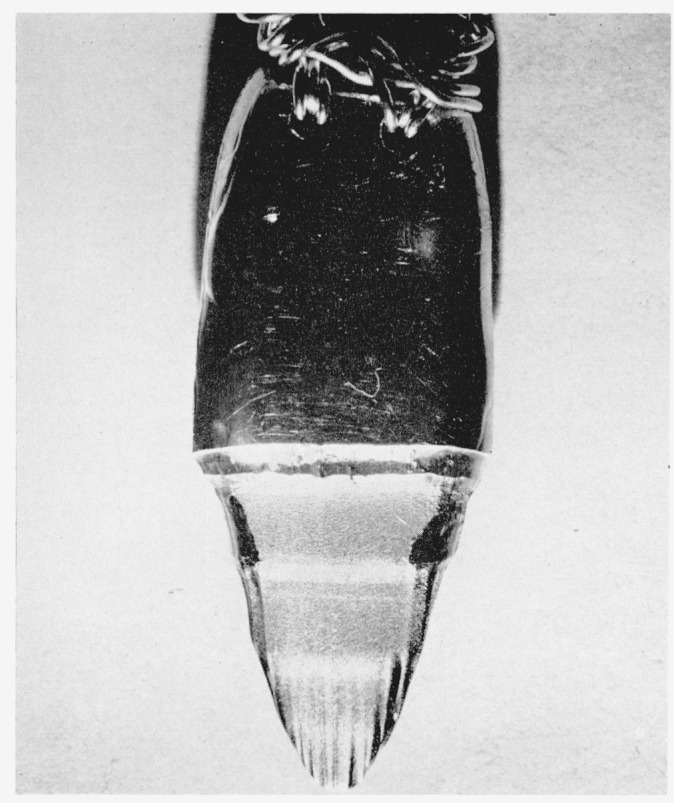
View of a silver anode showing the means of support and corrosion pattern.

Silver anodes of the elliptical rod form were supported in a different manner. Silver wires were inserted through holes bored in the upper end of the silver rods as shown in figure 4. These wires were looped through the holes several times and were covered with a sheet of silver foil at their upper parts. This silver foil was covered with a second foil and then clasped by gold-plated clips; the rest of the support was identical to that described above. Here also, the wires, silver foil, and gold-plated clips were above the solution.

In these arrangements the question may arise as to whether the electrolyte creeps up the anode and onto the silver foil and gold-plated clips during the electrolysis causing errors in the final results. Repeated checks were made on this possibility by weighing the silver foil over the period of time during which it had been used in experiments. Typical results on the constancy of the weight of the silver foil which was used with sheet anodes are given in table 1. No trend in the weight of the silver foil was observed over a period of 2 months. These data show that any creepage that occurred was insignificant. Similar results were found for the foil used with anodes of the rod type.

## 3. Coulometer Circuit

**Figure 5:**
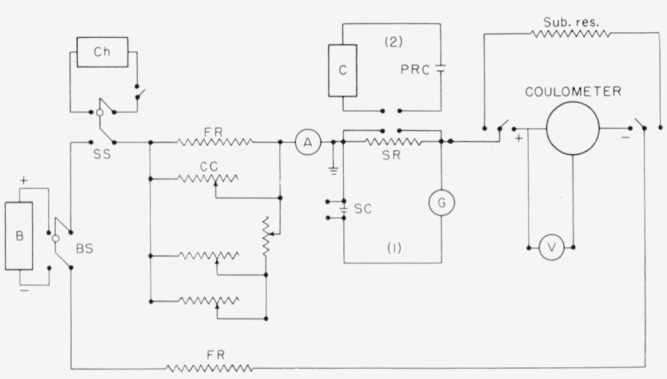
Coulometer circuit.

The circuit, sketched in figure 5, consisted of the coulometer with an alternate circuit of equal resistance, approximately 25 ohms (labeled Sub. res.) for use in adjusting the current prior to an actual run; a standard resistor (or resistors), SR; a battery, B; a double pole switch, BS, for connecting the battery to the circuit; two fixed resistors, FR; suitable rheostats, CC, for adjusting the current during the run. (These were in parallel with one of the fixed resistors, FR, which carried approximately 95% of the current.) The circuit also contained an ammeter, A; a chronograph, Ch; a reference silver electrode with a voltmeter, V (25,000 ohms), for measuring the potential of the silver anode during the electrolysis; and a large double-pole switch, SS, for simultaneously closing the coulometer and chronograph circuits. Two parallel circuits, 1 and 2, were used in measuring the *IR* drop across the standard resistors during the run.

## 4. Constancy and Magnitude of *IR*

The constancy of the *IR* drop across the NBS standard resistor or resistors was maintained by balancing the *IR* drop against the electromotive force of NBS saturated standard cell No. 1064 using a sensitive galvanometer as null detector (circuit No. 1). Thus, the *IR* drop should be equal to the electromotive force of the standard cell. The galvanometer key was always closed except when opened momentarily from time to time to check the zero. The galvanometer had a nominal sensitivity of 0.0007 *μ*a/mm of scale; actual experimental checks were made during each run and gave 3.5 ppm/mm for the current sensitivity at 0.2 amp for circuit No. 1. Circuit No. 2 consisted of NBS saturated standard cell No. 1073 (PRC) and a Brooks standard cell comparator [25]. Suitable leads and connectors were used so that the emf of cell No. 1064 and the potential across the standard resistors could be measured *independently* of one another.

Electromotive force readings on NBS standard cell No. 1064 (in circuit No. 1) were made prior to, during, and after a run using NBS saturated standard cell No. 1073 (in circuit No. 2) as the reference cell. These readings were made to verify that the emf of 1064 remained constant throughout the run. Illustrative data, obtained in a run on May 22, 1958, are given in table 2.

A direct and more accurate measure of the *IR* drop across the standard resistors was also made at intervals by means of circuit No. 2. With the Brooks comparator, in circuit No. 2, the actual *IR* drop across the standard resistors was measured against cell No. 1073. The sensitivity for these measurements was checked before each run and was 5 *μ*v/cm on the galvanometer scale. As an illustration, data taken on May 22, 1958 are given in table 3. The *IR* drop was 1.0182103 v which agrees exactly with that given by circuit No. 1 and no correction was necessary. In all cases the agreement was within 3 ppm; the *IR* drop as given by circuit No. 2 was always used in calculating the faraday.

Saturated standard cells No. 1064 and No. 1073 were in a well-stirred oil bath maintained during the run to ±0.002° C. The temperature of the bath during all the runs was 28.00°±0.01° C. The temperature of the bath was measured with a platinum resistance thermometer using a Mueller Temperature Bridge. Both cell No. 1064 and cell No. 1073 were compared prior to and alter a run with two cells m the National Reference Group (the group of standard cells used to maintain the volt for the United States).

Throughout a run frequent measurements were made of the potential of the silver anode against the reference electrode using the voltmeter, *V*, having a resistance of 25,000 ohms. The average number of coulombs used in these measurements for an entire run was only 0.0005 coulomb; this correction for each run was made, see footnote (^a^) of table 10.

Finally, a check was made on the time delay that might have occurred in the current reaching a steady value when the circuit was closed at the start of a run. This was done by connecting an oscilloscope across the standard resistor in a simulated experiment. When the starting switch was closed the oscilloscope indicated that a steady current was reached immediately.

In some runs, the initial current through the coulometer deviated very slightly from that desired; in these cases adjustments were quickly made and the coulombs were corrected accordingly. These corrections were almost negligible but are incorporated in the values of the coulombs given in tables 10, 15, and 17.

## 5. Magnitude of the Current

The NBS standard resistors were housed in a well-stirred oil bath and the temperatures of the resistors were measured at intervals throughout a run with calibrated mercurial thermometers placed in the central hole of the resistor coils. From the time-averaged temperature the values of the resistance of the standard resistors were calculated by known equations expressing the resistance of the coils as a function of temperature. These equations are of the form:

$$R_{t}=\left(nv+k\right)\left[1+\alpha \left(t-25\right)+\beta {\left(t-25\right)}^{2}\right]\text{ohms},$$

where *nv* (nominal value of resistor), *k, α*, and *β* have the values as given in table 4 for the seven different resistors used in this investigation. The temperature, *t*, is in degrees Celsius. Various combinations of the resistors were used; these will be discussed later. The resistance of t he standard resistors on the date the measurements were made were interpolated from the data for *k* given in table 4, the coefficients *α* and *β* did not change with time. The values, as calculated from the above equation and the data of table 4, are for conditions under which the heating due to the test current is negligible. Of course, during the coulometric experiments the current through the resistors is larger and part of the heat generated may alter the resistance of the standard resistors. In use, then, the load resistance rather than the nonload resistance of the standard resistors must be employed. These load resistances, as determined by the Resistance and Reactance Section of NBS, for currents of approximately 0.1, 0.2, 0.3, and 0.5 amp which were used in the runs, are given in table 5.

As shown in table 5, the load resistance differed from the nonload resistance only for high currents, i.e., 0.5 amp.

For illustration in the run of May 22, 1958, resistors 12 and 17 were used in parallel. Therefore, the magnitude of the current during the run was as given in table 6. The mean (i.e., time-averaged) current was, therefore, 0.20363887 amp which agrees exactly with the value obtained from the quotient of the mean *IR* drop and the mean resistance. In all cases the mean current agreed with the quotient of the mean *IR* drop and the mean resistance to within 0.1 ppm.

## 6. Time

**Figure 6:**
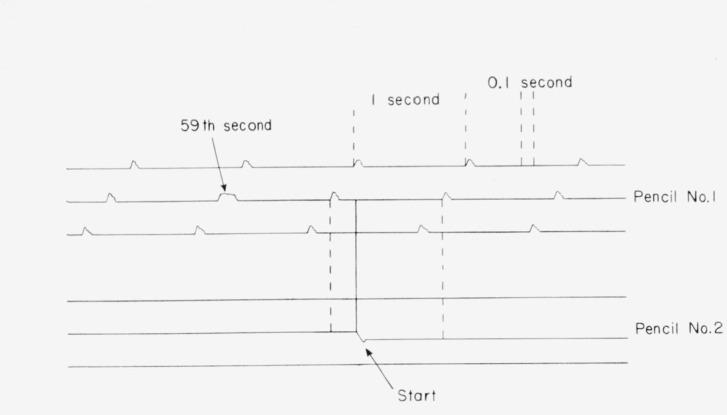
Illustration of recordings on chronograph chart.

The duration of a run was determined with the aid of an NBS chronograph. The second pulses were supplied by NBS standard crystal oscillators which keep mean solar time and the time of day was obtained from broadcasts of WWV. Since the 59th pulse is omitted in the NBS broadcasts from WWV the beginning of each minute could be identified with the time of day. The chronograph had two pencils, one (No. 1) was activated by the second signals (NBS crystal oscillators) by direct wire and indicates the start of each second on the chronograph chart; the 59th second has a characteristic trace. Pencil No. 2 was activated simultaneously with the opening or closing of the coulometer circuit by a double-pole switch (see fig. 5). This switch was closed after the signal from WWV had indicated the beginning of a new minute, and a displacement in the marking on the chronograph chart indicated the start or end of a run, as illustrated for a start in figure 6. A similar procedure was followed just prior to the end of a run. A radio receiver was used for picking up signals from WWV. The chronograph drum was started about 20 to 30 sec before the start and the end of a run.

Since the chronograph chart showed a constant distance, 7.48 cm, between the second markings the time could be ascertained to better than 0.01 sec. For a 3-hr run this means that the duration of a run was known to better than 1 ppm; for longer runs it was known, of course, with more accuracy.

To ascertain the delay in activating and deactivating the chronograph pencil when the switch was thrown, an electric timer used in the calibration of watthour meters and activated by a crystal-controlled 60-cps frequency was inserted directly in the coulometer circuit in several simulated starts and stops. Checks were made over 2-min time intervals. The electric timer was calibrated directly against NBS crystal oscillators. The total time indicated by the timer and the chronograph for a 2-min interval differed, on the average, by only 0.009±0.006 sec with the chronograph in all cases giving the lesser time. Thus, the correction used for the duration of a run was +0.009 sec. Although this correction is insignificant it was nevertheless applied in all the calculations.

## 7. Mass

For the runs with mint silver the masses of silver, crucibles, etc., were determined using a semimicro-balance. For those runs with purified silver rods a microbalance was used; its capacity was 20 g. Weighings were made by double transposition, with sensitivity measurements interspersed, in a constant-temperature room maintained at 25 ±1° C, and with a relative humidity of always less than 50 percent. The balance was supported on an Alberene slab anchored to a wall of the laboratory. All weighings were repeated until constancy was achieved. Silver sediment (see later) was collected in Pyrex crucibles and weighed using an identical crucible as a tare. The silver sediment was retained in the crucibles from one run to another. The densities of silver (10.50) and the laboratory air and also the densities of the brass (8.4), platinum (21.5), and aluminum (2.7) weights were used in making buoyancy corrections.

In calculating the density of the laboratory air the following data were used:

- Smithsonian Meteorological Tables, 6th revised edition, 1951, Smithsonian Institution Publication (specifically table 46). This publication gives data for correcting the barometric pressure at an observed laboratory temperature in degrees Celsius to the barometric pressure at 0° C.
- Psychrometric Tables for Obtaining the Vapor Pressure, Relative Humidity, and Temperature of the Dew Point, by C. F. Marvin, Weather Bureau, U.S. Department of Commerce, U.S. Government Printing Office, Washington, D.C., 1941. This publication gives the relation between relative humidity and the temperature difference, in degrees Fahrenheit, as given by a dry- and a wet-bulb thermometer.
- Design and Tests of Standards of Mass, NBS Circ. No. 3, 1918. Table 15 in this publication gives the data required for correcting the barometer for the observed relative humidity.
- National Bureau of Standards gravity = 980.100 cm sec^−2^; standard gravity at 45° latitude and sea level=980.665 cm sec^−2^.
- 1.293052 g liter^−1^ = density of dry air containing 0.04-percent carbon dioxide at 0° C and 1 atm, NBS Circ. No. 3, 79 (1918).
- 1/1.000028=factor to reduce values of milligrams milliliter^−1^ to milligrams cm^−3^.

The barometric pressure, temperature, and the humidity of the laboratory air were measured at the time of each weighing.

## 8. Materials and Purification

### 8.1. Materials

In the determination of the faraday by anodic dissolution of silver in aqueous solutions of perchloric acid containing a small amount of silver Perchlorate four materials were needed. These were: (1) silver, (2) perchloric acid, (3) silver Perchlorate, and (4) water. The silver used was high-grade samples received from the U.S. Mint in Philadelphia, Pa. It was stated by the mint that the silver was of high purity, had been purified by one or two electrolyses, was melted in silica, was rolled into sheet form on steel rolls, contained a trace of copper and perhaps lead, contained no occluded hydrogen, and was believed by the mint to be free of occluded and combined oxygen, unless allowed to stand exposed to the atmosphere.

The perchloric acid was reagent grade. The silver Perchlorate was prepared from reagent grade silver oxide and perchloric acid solution. Conductivity water was used in diluting the perchloric acid and in the necessary analytical operations [23]. It was produced in a conductivity still and one-third of the condensate was collected; the feed was distilled water treated with alkaline permanganate. The conductivity water had a conductivity of 0.6 to 1.0×10^−6^ (ohm cm)^−1^. The solution of perchloric acid (20 wt %) and silver Perchlorate (0.5 wt %) was slowly filtered through a fritted filter containing finely-divided silver prior to use in the coulometer. This precluded the possibility of the solution containing anions which would have precipitated silver ion during a coulometric run.

### 8.2. Purity of the Silver

Samples of two separate lots of mint silver were leached in concentrated hydrochloric acid solution and then in 10 percent aqueous ammonium hydroxide (extensive etching of the surface of the silver was deliberately avoided) to remove surface impurities. Both the etched and unetched samples^4^ were then analyzed spectrochemically. The results for the etched samples are given in table 7 and show that the purity of the two etched silvers was high; the total impurities in samples 1 and 2 were, respectively, 8.4 and 4.6 ppm, with copper being the highest in both cases. Since the copper percentage was relatively high sample No. 1 was analyzed photometrically. The silver was first dissolved in nitric acid and was then removed from the solution as the chloride. The copper remaining in the filtrate was determined by the carbamate-photometric method. The results agreed well with those obtained spectrochemically (see table 7). Since the etched samples dissolved completely in dilute nitric acid they were free of halides or sulfides.

Although the amount of metallic impurities was low in both samples of mint silver and methods were known for correcting for them, it was deemed advisable, nevertheless, to purify a sample of mint silver. Purification of silver posed quite a problem because, whether conventional chemical or electrochemical methods were used in the purification, the resulting material (silver) would be in granular or powder form unsuitable for use as electrodes. Not only would a purification be involved but also a process required wherein the silver could be made into electrodes without serious contamination. The methods by which this was achieved are described in the next section.

### 8.3. Purification of Silver

After considering the various methods described in the literature for the purification of silver it was decided that the electrolytic method was the most suitable for the present purpose. This method requires the preparation of a small quantity of only one pure reagent, viz., silver nitrate solution, and any occlusions of water or of electrolyte in the electrolytically deposited silver is readily expelled by subsequent vacuum fusion (the amount of occlusions is not significant, but complete expulsion is required). The electrolytic method thereby avoids the possibility of contaminating the final silver by occlusions of reagents which are not expelled by vacuum fusion and which are not detected by spectrochemical analysis. It may, of course, be said that the electrolytic method introduces the possibility that the deposited silver may be enriched with one or the other isotope of silver. However, as will be shown later, no measurable separation of the isotopes of silver occurred in this process. Furthermore, the atomic weight of the silver used in this investigation was determined in an associated experiment (see later).

The electrolytic cell used in purifying the silver consisted of two beakers containing silver nitrate solution joined by a siphon with a glass frit sealed halfway between the legs of the siphon. This silver nitrate solution was prepared from a small quantity of recrystallized silver nitrate. A stopcock was sealed to the top of each leg and on each side of the frit so that both legs of the siphon could be readily filled by suction after silver nitrate solution was poured into each beaker. The frit prevented mixing of the anolyte and catholyte and also migration of particles. Both electrodes were bars of mint silver having a cross section of 4.0×0.25 cm and were about 10 cm in height. The bulk of the purified silver came by the mass transport from the anode to the cathode and the initial silver nitrate solution served only for initial conductivity. The silver bars were part of a third sample obtained from the Philadelphia Mint and had a purity less than that of the first two samples; total impurities amounted to about 120 ppm. During the electrolysis a little silver oxide was added at intervals to the anode beaker to keep the anolyte slightly basic thereby precipitating many metallic impurities as the oxides. Also during the electrolysis a little concentrated nitric acid was added at intervals to the cathode beaker to keep the catholyte acidic whereby the deposition of silver was favored over that of metallic impurities. The electrolysis was carried out in a closed cabinet to minimize exposure of the electrolytic cell to dust and fumes.

At intervals the cathode was removed from the cell and the deposited silver was easily scraped from the underlying silver cathode into a Pyrex beaker by a silver spatula. Each portion of silver so removed was kept in a separate beaker containing dilute nitric acid. The beakers were covered and kept in a closed cabinet until the silver was used to prepare anodes. Most of the silver deposited as clusters of glistening crystals; the remainder was in the form of needles. Spectrochemical analyses showed no significant difference in the purity of the two forms. Typical results of spectrochemical analyses of samples of the finely-divided silver are given below:

**Table:** 

Sample No.	Impurities detected
	
1	<1 ppm Mg
2	<1 ppm Mg
3	<1 ppm Mg and <1 ppm Cu 1–10 ppm Si
4	none
5	0.01 ppm Mg and 0.01 ppm Ca
6	$\left\{ \begin{array}{l} 0.01\;\text{ppm Mg and}\;0.01\;\text{ppm Ca}\\ 0.05\;\text{ppm Al} \end{array}\right.$

### 8.4. Preparation of Pure Silver Anodes

When a portion of the electrolytic silver was needed to prepare an anode, the dilute nitric acid was decanted and the silver transferred to a polyethylene container which had been soaked in hydrofluoric acid solution. The silver was then covered with fresh hydrofluoric acid solution and allowed to stand for at least 2 weeks to remove any silica that may have been attached to or embedded in the surface of the silver during its purification. The hydrofluoric acid was then decanted and the silver washed with conductivity water. The silver was allowed to stand under conductivity water for a period of at least 2 weeks with frequent changes of the water and then transferred to a perforated silver dish and dried at 110° C for several hours in an oven.

The dried and glistening silver was then transferred to a transparent fused silica tube which had been inserted and sealed into a cylindrical opening in the detached flange of the head of a high-temperature furnace. Before sealing the silica tube to the detached flange the tube was closed at one end and dented and then leached in hot concentrated nitric acid, well rinsed with conductivity water, and dried in an oven. “O” rings and a suitable wax were used in sealing the tube into the flange. With the tube sealed in the flange and in a vertical position the space between the bottom of the tube and the dent was filled with the finely-divided silver. The flange was then attached to the furnace head with the tube in a horizontal position. The dent in the tube served to restrict the flow of the silver when it melted.

When the furnace was operating the head was cooled by circulating water thereby keeping the wax seal and the “O” rings cool. The furnace was of the resistive type and was provided with automatic programing equipment, temperature recorder, a mechanical pump, a diffusion pump, a pressure gauge, and a cold trap. After the silver was melted the furnace was allowed to cool and the silver to solidify. When the temperature was well below the melting point of silver the heating elements were pulled back and the tube was allowed to cool more rapidly. This procedure caused the tube to crack where it was in contact with the solidified silver and finally the silver fell from the tube and was caught with a towel. The cross section of the silver rods, thus produced, was approximately elliptical. Some pieces of the silica tube adhered to the silver when it fell from the tube. No attempt was made to remove any adhering silica mechanically; this was removed by soaking the silver in hydrofluoric acid solution and its complete removal was judged by the constancy of the weight of the silver with repeated treatment with fresh hydrofluoric acid. The same furnace was used in the heat treatment of the sheet (mint) silver. The heat treatment of the sheet silver and the melting of the purified silver is described below.

Samples were then cut from the silver for spectrochemical analysis and two small holes were then drilled in one end (upper) of the silver and silver wire was entwined through these holes (see fig. 4). This wire served to suspend the silver in the analytical balance and also for an electrical connection. The constancy of the weight of the anode after prolonged soaking in hydrofluoric acid was again checked; this check was followed by soaking the anode first in 10-percent aqueous ammonium hydroxide and then in the perchloric acid-silver Perchlorate solution. Since silver is remarkably stable in the latter solution etching ensued only in the ammonium hydroxide solution (appreciable etching was purposely avoided, as before for mint silver).

In all, five different anodes were made according to the above general procedure; in each case, the anode was prepared with some modification in the general procedure and these will be outlined in the next subsection. Spectrochemical analyses of the anodes, anode No. 3 excepted, are given in table 7. As will be seen, the anodes were of very high purity and the extremely small amounts of impurities are inconsequential in a faraday determination (correction for these impurities was 1 ppm, see later). As will be discussed in appendix 2, anode 3 was prepared by melting silver in an atmosphere of hydrogen in a silica tube. This procedure caused the silver to blister and to absorb relatively large amounts of impurities from the silica tube. Accordingly, anode 3 was not used in determining the value of the faraday.

### 8.5. Treatment of the Silver

It is well known that silver has an affinity for oxygen. Steacie and Johnson [26] in a comprehensive study of the silver-oxygen system found that the solubility of oxygen in silver is proportional to the square root of the oxygen pressure and exhibits a minimum solubility at about 400° C at each pressure. They concluded that oxygen existed as the element in silver above 400° C and combined with silver as the lower oxide, Ag_2_O, below 400° C. Since Ag_2_O is readily soluble in perchloric acid its existence in metallic silver would lead to appreciable errors in a faraday determination since the dissolved Ag_2_O would bear no relation to the quantity of electricity passed through the coulometer.

Fortunately, Ag_2_O readily decomposes on heating, and as early as 1888 Carnelly and Walker [27] showed the decomposition of Ag_2_O is complete between 300° and 430° C. Lewis [28], Keyes and Kara [29], and Benton and Drake [30] measured the decomposition of Ag_2_O over a temperature range of 173° to 500° C with the results given in table 8. By an extrapolation of Ins data Lewis calculated the decomposition pressure of silver oxide to be 5×10^−4^ atm at 25° C. In a recent electrochemical study of the Ag,Ag_2_O electrode Hamer and Craig [31] obtained 2,723 cal for the free energy of decomposition of Ag_2_O at 25° C; this value corresponds to 0.988×10^−4^ atm for the decomposition pressure of Ag_2_O at 25° C.

Since oxygen exists at a partial pressure of about 0.21 atm in air (air contains about 21% oxygen) at NTP it is apparent by extrapolation of the data in table 8 that silver must be heated above 150° C in an open system to effect the decomposition of any Ag_2_O it may contain. However, heating to temperatures above 150° C in an open system will not insure the removal of all silver oxide (or oxygen) since some oxygen will remain or redissolve in the silver to maintain the solubility relations given by Steacie and Johnson (see appendix 1). Therefore, the oxygen, as it is formed in the decomposition of the silver oxide, must be removed from the system. If oxygen remains in the silver it will, of course, affect the apparent value of the faraday because it would be included in the weight of the silver dissolved.

The oxygen formed in the decomposition of Ag_2_O may be removed either by evacuation or by heating the silver in hydrogen. In the latter case the gaseous water formed in the reaction:

$$H_{2}\left(\text{gas}\right)+\left(½\right)\;O_{2}\left(\text{gas}\right)\to H_{2}O\;\left(\text{gas}\right)$$

and all hydrogen remaining must be removed either by evacuation or by complete displacement with an inert gas. Hydrogen should be removed for it may reduce silver ions formed in the electrolytic dissolution of silver back to metallic silver causing errors in the faraday. Water must be removed since its presence will cause errors in determining the weight of the silver dissolved.

In view of the above considerations the silver, whether it was mint silver No. 1, mint silver No. 2, or electrolytically purified silver, was given special treatments designed to completely decompose the silver oxide and remove all of the oxygen from the starting silver. These treatments were as follows.

#### a. Mint Silver No. 1

##### Experiments 1, 2, and 3

Silver used in these experiments was from one sheet. The sheet was treated with hydrogen in a clear silica tube at 862° C (98° C below the melting point of silver) for 2 hr. The hydrogen was then displaced slowly by helium over a period of 118 hr with the temperature maintained at 862° C. The tube containing the silver and helium was then cooled to 540° C in 3 hr and finally to room temperature in an additional hour. The silver was then removed from the tube, etched in 10-percent aqueous ammonium hydroxide, washed with conductivity water, and dried at 110° C in an oven.

##### Experiments 4, 5, and 6

Silver used in these experiments was from one sheet. The sheet was treated with hydrogen in a silica tube at 860° C (100° C below the melting point of silver) for 96 hr or considerably longer than samples used in experiments 1, 2, and 3. The hydrogen was then displaced slowly by helium over a period of 250 hr with the temperature maintained at 860° C. The tube containing the silver and helium was then cooled slowly to room temperature. The silver was then removed from the tube and treated in the same manner as the samples used in the preceding experiments.

#### b. Mint Silver No. 2

##### Experiment 7

The silver used in this experiment was heated in *vacuo*, 5*μ*, in a silica tube at 500° C for 1 hr and then cooled in *vacuo* to room temperature in a little over an hour. The silver was then removed from the tube and treated in the same manner as that used in the preceding experiments.

##### Experiments 8, 9, and 10

Silver used in these experiments was from one sheet. The sheet was heated in *vacuo*, 2*μ*, in a silica tube at 500° C for 5 hr and then cooled in *vacuo* to room temperature in a little over an hour. The silver was then removed from the tube and treated in the same manner as the samples used in the preceding experiments.

##### Experiments 11, 12, and 13

Silver used in these experiments was from one sheet. The sheet was heated in *vacuo*, 3*μ*, in a silica tube at 500° C for 10 hr and then cooled in *vacuo* to room temperature in a little over an hour. The silver was then removed from the tube and treated in the same manner as the samples used in the preceding experiments.

##### Experiments 14 and 15

Silver used in these experiments was from one sheet. The sheet was placed in a silica tube which was then evacuated to 50*μ* in 1 hr. Helium was then introduced for 30 min and then the tube evacuated. Hydrogen was then introduced into the tube which was then heated to 860° C over a 15-hr period. The tube containing the silver and the hydrogen was then kept at 860° C (100° C below the melting point of silver) for 96 hr and then cooled to room temperature in 1 hr. The silver was then removed from the tube and treated in the same manner as the samples used in the preceding experiments.

##### Experiments 16 and 17

The silver used in these experiments was of one sheet. The sheet was placed in a silica tube which was then evacuated to 200*μ* in ½ hr. Helium was then introduced and the temperature raised to 860° C in 3 hr and then flushed with hydrogen. The tube containing silver and hrydrogen was kept at 860° C for 101 hr and then cooled to room temperature in 1 hr. The silver was then removed from the tube and treated in the same manner as samples used in the preceding experiments.

#### c. Purified Silver

##### Experiments 18, 19, and 20 (anode No. 1)

One rod or anode of silver was used in these three separate experiments. The finely-divided silver was heated in *vacuo* in a silica tube to 975° C (15° C above the melting point of silver) ; the rate of heating from room temperature was 100° C/hr. The silver was held in the molten state at 975° C for ½ hr in *vacuo*, 0.7 *μ*, and then cooled in *vacuo*, 0.7 *μ*, until the tube cracked. The silver was then removed from the tube and placed in a solution of hydrofluoric acid to remove any attached quartz as discussed in section 8.4. The anode was then etched slightly in 10-percent aqueous ammonium hydroxide, soaked for a short time in perchloric acid-silver Perchlorate solution, washed with conductivity water, and dried at 110° C in a confined space. The anode was then ready for use.

##### Experiments 21, 22, 23, 24, and 25 (anode No. 2)

One rod or anode was used in these five separate experiments. The finely-divided silver was heated in a silica tube to 975° C for 1 hr in *vacuo*, 0.5 *μ*, and then cooled in *vacuo* until the tube cracked. The silver was then removed from the tube and treated in the same fashion as the preceding anode.

##### Experiment 26 (anodes 1 and 2 combined)

Portions of anodes 1 and 2 which were left after the above experiments (18 to 25, inclusive) were combined for this experiment.

##### Experiments 27 and 28 (anode 4)

One rod or anode was used in these two separate experiments. The finely-divided silver was placed in a silica tube which was then evacuated at room temperature over a 2-hr period to 1 *μ*. Hydrogen was then introduced and the tube heated to 500° C in 5 hr, and kept at this temperature overnight. The hydrogen was then displaced by helium in 1 hr and the tube evacuated to 1 *μ.* The evacuated tube containing the silver was then heated to 975° C for 1 hr at 0.7 *μ* and then cooled in *vacuo* until the tube cracked. The silver was then removed from the tube and treated in the same manner as the preceding anodes.

##### Experiments 29, 30, and 31 (anode 5)

One rod or anode was used in these three separate experiments. The finely-divided silver was placed in a silica tube which was then evacuated at room temperature over a 4-day period to 50 *μ.* Hydrogen was then introduced into the evacuated tube which was then heated at 500° C in 5 hr and held at 500° C for an additional 3 hr. The tube was then evacuated to 70 *μ* overnight, further evacuated to 0.9 *μ* in 2 hr and finally heated to 975° C. The tube with the molten silver was held at 975° C for 1 hr at 0.9 to 0.7 *μ* and then cooled to 895° C (65° C below melting point of silver) over a 20-min period (i.e., through the solidification period) and finally cooled in *vacuo* until the tube cracked. The silver was then removed from the tube and treated in the same manner as the preceding anodes. X-ray measurements showed this anode had a single crystal orientation.

The treatments discussed above may be summarized as follows: (1) All mint silver was heated *below* the melting point of silver while all anode silver was heated to 15° C *above* the melting point; (2) mint silver was heated in hydrogen, hydrogen and helium, or *vacuo* for various periods of time; and (3) anode silver was heated in *vacuo*, in hydrogen, or in hydrogen and helium for various periods of time. The effect of these various treatments are discussed in section 11.

## 9. Procedure

### 9.1. General Procedure

In the foregoing sections some of the procedures, particularly those dealing with the maintenance and constancy of the electric current and measurements of mass and time, were outlined. In this section, manipulative procedures involved in actual determinations of the electrochemical equivalent of silver will be described. Mint silver in sheet form, or purified silver in rod form, after being accurately weighed was suspended in a filtered aqueous solution of perchloric acid (20-wt %)-silver Perchlorate (0.5-wt %) contained in a Pyrex beaker by the arrangement shown in figure 3. The coulometer and chronograph circuits were then closed simultaneously and a controlled electric current was passed through the coulometer for several hours. At the end of the run, the silver sheet or rod was removed, washed with conductivity water, dried at 110° C, and weighed.

As the silver goes into solution some particles (sediment) fall to the bottom of the anode beaker. This sediment was collected in a weighed Pyrex crucible with a fritted bottom, repeatedly washed with conductivity water, dried at 110° C, and weighed. This weight of the sediment was then subtracted from the difference between the initial and final weights of the sheet or rod to give the weight of the silver which was electrolytically dissolved (see later). Since the coulometer was open so that the silver sheet or rod could be lowered as it dissolved the possibility existed that the solution might be contaminated with dust, even though the entire coulometer was housed under a plastic cabinet. Accordingly, another Pyrex beaker containing the coulometer solution in an amount equal to that used in the anode beaker was placed uncovered beside the anode beaker. This solution was not a part of the coulometer but served as a blank. At the end of a coulometer run this beaker was covered with an inverted beaker. A day later this solution was filtered through the same fritted crucible used to collect the silver sediment. The change in weight of the fritted crucible for the blank solution was always small, nearly negligible, but nevertheless was used as a blank correction for the weight of silver sediment. In the early runs with mint silver an average blank correction was used; for the later runs with purified silver anodes the blank correction was obtained for each individual run.

### 9.2. Variation in Conditions

In all, 31 quantitative and 22 informative measurements were made. The latter are discussed in the appendices. The conditions of the experiments were varied as follows:

- *The current density, i.e., amperes per square centimeter of anode surface, was varied.* This was accomplished either by using different areas of exposed silver in the coulometer solution or by passing currents of different magnitudes through the coulometer. The different magnitudes of current are listed in table 9. The current density for both the mint silver anodes and the purified silver anodes was varied from 0.025 to 0.150 amp/cm^2^ and no trend in the value of the faraday was found. The resistors used, their assembly, and a code for the assembly (for use in tables 10, 15, and 17) are also given in table 9.
- *The potential of the silver anode during the electrolysis was varied.* This change resulted from the changes outlined in (a) above. When the electrode areas were decreased or the current increased the potential of the corroding anode, relative to the reference electrode, became higher. The average values of the silver anode potentials, *E*_r_, during each run are given in table 10. These values ranged from 0.088 to 0.201 v for the mint silver anodes and from 0.089 to 0.149 v for the purified silver anodes.
- *The duration of the coulometric runs and the amount of silver dissolved were varied.* The shortest time was 2 hr, the longest 6½ hr. The least amount of silver dissolved in any one run was 2.4 g, the most 5.0 g.
- As stated in preceding sections *different samples of silver were used.*
- As stated in preceding sections the *samples of silver were treated in various ways.*

## 10. Electrophoresis of Silver

Dining a run the possibility existed that some of the silver sediment may have been so finely divided that it might have electrophoretically migrated toward the cathode, and thereby be removed from the anode beaker. To check if such were the case after the siphons were drained the solution in the beaker adjacent to the anode beaker (see fig. 1) was carefully filtered through the Pyrex crucible after many runs. No significant difference was found in any experiment between the weight of the sediment contained in this beaker and the weight of the sediment in the blank beaker. Thus, no silver sediment was transferred from the anode beaker by electrophoresis, or any other mechanism.

## 11. Results

### 11.1. Electrochemical Equivalent of Silver

The experimental quantity obtained directly from the coulometric measurements is the electrochemical (or coulometric) equivalent of silver. In table 10 the results of the 31 quantitative experiments are given. The headings of each column are in general self-explanatory. Column 10 gives the quantity of silver anodically dissolved by the coulombs given in column 8. Column 11 gives the amount of sediment produced in each run; it was used to obtain the values given in column 10. It will be noted that the amount of sediment produced varied widely and was extremely small in the last 5 runs made with purified anodes; the last and perhaps all anodes had a single crystal orientation. In some cases lumps of silver fell from the anode during the electrolysis; for these see runs No. 3, 7, 14, 15, 16, 17, and 20. Even though the amount of sediment differed widely between the various runs this variation did not affect the final results as is apparent from the concordance obtained (this fact also shows that the sediment could not have consisted predominantly of one or other of the two isotopes of silver, see page 382). Column 9 gives the average potential of the silver anode during the electrolysis. A high value means either that the anode surface immersed in the solution was smaller or the magnitude of the current higher than in those runs in which the potential was lower. Inspection of the values in column 13 show that they are independent of the values of the anode potentials, *E_r_.*

The values of the electrochemical equivalent of silver in the next to last column of table 10 are based on the simple relation:

$${\text{Ag}}_{\text{initial}}^{\text{bulk}}-{\text{Ag}}_{\text{final}}^{\text{bulk}}-\text{sediment}={\text{Ag}}_{\text{measured}\left(\text{or dissolved}\right)}={\text{Ag}}_{m}$$ (1)

and are, therefore, uncorrected for the minute amounts of metallic impurities in the silver. Actually, in these calculations the quantity ${\text{Ag}}_{\text{electrolytic}}^{\text{pure}}$ should be used rather than Ag*_m_* in determining the electrochemical equivalent of silver and is obtained from values of Ag*_m_* by the relation:

$${\text{Ag}}_{\text{elcetrolytic}}^{\text{pure}}={\text{Ag}}_{m}+{\text{Ag}}_{\text{precipitated}}-\Sigma \prime i={\text{Ag}}_{m}\left(1+X-Y\right).$$ (2)

In this relation Ag_precipitated_ and Σ′*i* are given, respectively, by *X* Ag*_m_* and *Y Ag_m_* where *X* is the summation of the percentage of each metal impurity multiplied by the respective metal-ion valence and *Y* is the summation of the percentage of each metal impurity other than silicon which must exist as SiO_2_.^5^

The derivation of eq (2) follows: The metal impurities in the silver when released during the dissolution of silver will reduce Ag^+^ ions formed during the electrolysis to metallic silver according to the general reaction:

$$\text{Metal}+\gamma {\text{Ag}}^{+}\to {\text{Ag}}_{\text{precipitated}}+M^{\gamma +}$$

where *γ* is the valence of the metal-ion impurity, and thus the electrochemical equivalent of each metal is used. This silver will become part of the sediment. If, however, the metallic impurities are directly oxidized electro chemically, no error arises in these calculations assuming the above mechanism. The SiO_2_ released from the silver during the electrolysis will become part of the sediment. Also any other inert material, Σ*i*_inert_ (Pd or Au, for example in mint silver No. 1) released as the silver is electrolytically dissolved will also become part of the sediment. Accordingly, then, the sediment is given by:

$$\text{Sediment}={\text{Ag}}_{\text{sediment}}^{\text{bulk}}+{\text{Ag}}_{\text{precipitated}}+{\text{SiO}}_{2}+\Sigma i_{\text{inert}}.$$ (3)

Substitution of (3) in (1) gives:

$${\text{Ag}}_{\text{initial}}^{\text{bulk}}-{\text{Ag}}_{\text{final}}^{\text{bulk}}-{\text{Ag}}_{\text{sediment}}^{\text{bulk}}-{\text{Ag}}_{\text{precipitated}}-{\text{SiO}}_{2}-\Sigma i_{\text{inert}}={\text{Ag}}_{m}$$ (4)

or

$${\text{Ag}}_{\text{electrolytic}}^{\text{bulk}}-{\text{Ag}}_{\text{precipitated}}-{\text{SiO}}_{2}-\Sigma i_{\text{inert}}={\text{Ag}}_{m}.$$ (5)

Now,

$${\text{Ag}}_{\text{electrolytic}}^{\text{bulk}}={\text{Ag}}_{\text{electrolytic}}^{\text{pure}}+{\text{SiO}}_{2}+\Sigma \prime i+\Sigma i_{\text{inert}}$$ (6)

since the bulk silver that goes into solution during the electrolysis is equal to the sum of the pure silver electrolyzed, the SiO_2_ released, and the impurities other than SiO_2_ released. Substitution of (6) in (5) Leads to eq (2) since SiO_2_ and Σ*i*_inert_ cancel. In making corrections for the impurities they were taken as metallic except when the solid silver was only superficially heated in hydrogen or in *vacuo* for a very short time in which case they were taken as the oxide.

Final values of the electrochemical equivalent of silver, after corrections were made for the presence of trace metallic impurities, are given in the last column of table 10. Corrections for impurities are very small, in fact for the purified silver anodes the corrections amount to only 1 ppm or less. For mint silver No. 1 the average correction is 16.5 ppm while for mint silver No. 2 the average correction is 9.6 ppm.

The average value for all the mint samples is 1.117962 $\left(S_{\bar{x}}=5.1\times 10^{-6}\right)$ whereas the average value for all the purified silver anodes is $1.117968\;\left(S_{\bar{x}}=5.7\times 10^{-6}\right)$. The average of all values is $1.117965\;\left(S_{\bar{x}}=3.8\times 10^{-6}\right)$. $S_{\bar{x}}$ designates the estimated standard deviation of the mean.^6^ The agreement between the mint and purified samples is good. These averages, however, do not give a complete picture of the results. If instead, the average of the hydrogen values (silver treated with hydrogen) and the average of the vacuum values (silver heated or melted in *vacuo*) are tabulated, the following comparison is obtained:

**Table:** 

	Hydrogen	$S_{\bar{x}}\times 10^{6}$	Vacuum	$S_{\bar{x}}\times 10^{6}$	Difference
					
Mint silver	1.117953 (10)^a^	6.7	1.117974 (7)	8.0	0.000021
Purified silver	1.117960 (5)	9.5	1.117972 (9)	7.0	.000012
All values	1.117956 (15)	5.5	1.117973 (16)	5.3	.000017

a Numbers in parentheses equal number of runs.

The hydrogen value is lower than the vacuum value for both mint and purified silvers. The difference is statistically significant at the 0.05-probability level,^7^ and furthermore, this is most important, the hydrogen values are lower. This agrees with the fact that the electrochemical equivalent of hydrogen is considerably less than that of silver and, therefore, its presence could result in a lowering of the experimental value of the electrochemical equivalent of silver.

In view of the above comparison a more detailed comparison of the individual treatments given the silver was made and is outlined in table 11. Of the five distinct groups of values given in table 11 (3 for hydrogen and 2 for vacuum), the value of group 5, the last vacuum group, is 1.117972 mg coulomb^−1^. For this group, the silver had been purified, *melted* in *vacuo*, the impurity corrections were almost negligible (1 ppm or less), and no foreign substance, viz, hydrogen or helium had been used in heat treatment. The data for the hydrogen groups show that the longer the silver was treated with hydrogen the lower was the measured value of the electrochemical equivalent of silver even though attempts had been made to remove the hydrogen (group 2, excluded) ; no exception was noted in the three cases. On the other hand, no definite trend with time was found in the vacuum values for either mint or purified silver (the purified samples were melted in silica tubes but were not kept in the molten state for extended periods of time because of the danger of contaminating the samples. Previous to melting only a relatively small portion of the silver was in contact with the silica tube). Because of the trend in the hydrogen values they were all extrapolated linearly to zero time of hydrogen treatment. Since for mint silver No. 2 the times of treatment were so nearly identical, the correction for 96 hr obtained for mint silver No. 1 was used to correct mint silver No. 2 for both 96 and 101 hr. The average values obtained after these corrections are as follows:

**Table:** 

Silver	Number of runs	Extrapolated value	
			
			$S_{\bar{x}}\times 10^{6}$
Mint No. 1	6	1.117968	12.5
Mint No. 2	4	1.117969	20.8
Purified	5	1.117977	10.0
Average	15	1.117971	10.7

The average value 1.117971 agrees very well with the value, 1.117972, for the last vacuum group, group 5 in table 11. The average of all the vacuum and all the hydrogen corrected values is $1.117972\;\left(S_{x}=5.9\times 10^{-6}\right)$. For completeness the final individual values of the electrochemical equivalent of silver corrected for metallic impurities and residual hydrogen are given in table 12. The electrochemical equivalent of silver is, therefore, **1.117972 mg coulomb**^−1^ with an overall uncertainty of 0.000019 which includes 0.000012 as the limit to the effect of random errors (see bottom of table 12) and 0.000007 for the effects of known sources of possible systematic error.

### 11.2. Atomic Weight of Silver

Since the faraday is given by the ratio of the atomic weight to the electrochemical equivalent of silver it is necessary to know, or determine, the atomic weight of the silver used in the faraday determinations. The international value for the chemical atomic weight is 107.880 [32]. There is abundant evidence that the isotopic composition of natural occurring silver is nearly constant [12 and 13, also 33]. Using this value for the atomic weight of silver and 1.117972 mg coulomb^−1^ for the electrochemical equivalent of silver one obtains 96496.16 coulombs g-equivalent^−1^ and 96522.70 coulombs g-equivalentr^−1^ for the faraday on the chemical and physical scales, respectively; the factor 1.000275 being used in the conversion [32].

The chemical value 107.880 is based primarily on the experimental ratio AgNO_3_/Ag using the chemical atomic weights of oxygen and nitrogen (14.008). If the value of the atomic weight for nitrogen (14.00733) as calculated from nuclidic masses and abundances, measured by mass spectrometry [34], is used, an average value for the atomic weight (chemical scale) of 107.8771 is obtained. If the atomic weight of silver is calculated from the most recent work on the I/Ag ratio, assigning the mass spectrometer value to iodine (126.9101), the atomic weight of silver is 107.8770.

**Table:** 

Ratio	Ratio value	Atomic weight of silver chemical scale	Reference
			
AgNO_3_/Ag	1.574801	107.8762	[12]
AgNO_3_/Ag	1.574791	107.8781	[13]
I/Ag	1.176433	107.8770	[35]
		
Average		107.8771	

This average value then leads to 96493.56 coulombs g-equivalent^−1^ and 96520.10 coulombs g-equivalent^−1^ for the faraday on the chemical and physical scales, respectively.

Since it is the value of the faraday on the physical scale that is of most concern to those interested in atomic constants a direct determination of the physical atomic weight of silver was deemed most important. Accordingly, the physical atomic weight of silver was determined by mass spectrometry in an associated experiment by W. R. Shields and V. H. Dibeler of the Mass Spectrometry Section of the National Bureau of Standards. Their results are described in detail in reference [33]. They also measured the isotope ratio, Ag^107^/Ag^109^, in the mint and electrolytically purified silver anodes used in the present investigation and obtained 1.08148 uncorrected for instrumental bias. This ratio agreed with the ratio obtained for the silver in certified reagent grade silver nitrate and, within the experimental uncertainty, with the ratio obtained for native silver from various sources [33]. The agreement obtained between mint silver and the electrolytically purified silver is conclusive evidence that no measureable isotopic separation had occurred in the electrolytic process used to purify the silver for the anodes. Incidentally, these measurements also show that no separation of isotopes was likely during silver deposition in the earlier experiments of Rosa, Vinal, and McDaniel [21] or of Vinal and Bates [8,9].

In order to measure the bias, isotopieally enriched silvers from the Oak Ridge National Laboratory at Oak Ridge, Tenn., were used. The silvers as obtained were stated to have the isotopic composition as outlined in table 13. The samples were also measured by Shields and Dibeler at the National Bureau of Standards and their results agreed closely with those of the Oak Ridge National Laboratory.

Synthetic blends of weighed quantities of the isotopically enriched silvers were prepared and their isotope ratios were calculated using the calculated atomic weights of each enriched silver. The nuclidic masses used for Ag^107^ and Ag^109^ were, respectively, 106.93899 ±0.00010 and 108.93928 ±0.00010 [36]. Finally, the isotope ratios were measured with the mass spectrometer [33] and the results are given in table 14. In the last two experiments the synthetic blends were made with Ag^107^ and Ag^109^ which were purified electrolytically by the same procedure used to prepare the pure silver anodes used in the coulometer experiments with a few minor modifications needed because of the small samples of isotopieally enriched silvers that were available. Small silver rods of the enriched silvers were made as in section 8.4 and used as cathodes on which to deposit the purified samples.

Silver nitrate solution prepared from each isotopically enriched silver was used as the electrolyzing solution in each case. After deposition the purified silver was easily removed from the cathode and was leached in hydrofluoric acid solution and then in conductivity water. After it was dried at 110 °C, the silver was put in a small well-cleaned quartz tube and melted in *vacuo.* The resulting buttons were again leached in hydrofluoric acid solution and in conductivity water. Portions of each button were used to prepare synthetic mixtures, and other portions were analyzed spectrochemically. These analyses gave the following:

**Table:** 

Impurity	Ag “107”	Ag “109”
		
	*ppm*	*ppm*
Copper	0.05	0.05
Iron	.20	.20
Magnesium	.02	.05
Tin	.10	.5
	
Total	.37	.80

The total impurities in each case were less than 1 ppm; the two silvers were, therefore, of almost identical purity and the small difference would be without significant effect on the calculated or measured isotope ratios.

Originally, each of the two silvers contained about 125 ppm of total impurities. The results shown in table 14 for the measured isotope ratios show quite clearly that the impurities in the silver, providing they are of about the same amount in both silvers, do not affect the mass spectrometer determinations.

The weighted average of the bias ratio between the calculated and the measured isotope ratios (Ag^107^/Ag^109^) was 0.99467. Therefore, the mass spectrometer is biased in favor of the light isotope. However, this bias must also be taken into account in the values obtained for the analysis of silver “107” and “109”. When this is done for four successive calculations, the analyses of the original enriched materials are as shown in table 13. Using these analyses of silvers “107” and “109” the isotope ratios were recalculated. These are given in the second portion of table 14; the weighted average bias between the calculated and measured ratios is now 0.99444. Hence the true isotope ratio for the “faraday silver” is 1.07547. This value is equivalent to

107.9028±0.0013=at.wt.of silver(physical scale)

and

107.8731±0.0013=at.wt.of silver(chemical scale)

The uncertainty of 0.0013 comes from the assigned uncertainty in the nuclidic masses of Ag^107^ and Ag^109^ and the statistical uncertainty (95% confidence level) in the mass spectrometer measurements. No additional uncertainty is assigned in conversion to the chemical scale since the adopted conversion factor is taken as 1.000275 exactly.

### 11.3. The Faraday

From the above determinations of the electrochemical equivalent of silver and the atomic weight of silver measured with the mass spectrometer, the faraday is

$$F=96,516.5\pm 2.4\;\text{coulombs}\;g\text{-equivalen}t^{-1}\;(\text{physical}\;\text{scale}).$$

^8^

Using the conversion factor, the faraday on the chemical scale is

$$F=96,490.0\pm 2.4\;\text{coulombs}\;g\text{-equivalen}t^{-1}\;(\text{chemical}\;\text{scale})$$

where the uncertainties include both the uncertainties in the electrochemical equivalent and atomic weight of silver.^9^

## Figures and Tables

**Table 1:** Data on the constancy in weight of silver foil used in the support of silver anodes

Date	Hour	Electrolyses	Weight of silver foil (after electrolyses)
			
*1956*			*g*
Aug. 27	4:15 p.m.	None	1.937572
Aug. 29	10:00 a.m.	One	1.937568
Aug. 30	4:00 p.m.	One	1.937556
Aug. 30	8:15 p.m.	None	1.937568
Sept. 16	7:55 p.m.	None	1.937567
Sept. 17	4:00 p.m.	One	1.937558
Sept. 19	4:10 p.m.	One	1.937553
Sept. 23	9:50 p.m.	None	1.937554
Sept. 25	2:15 p.m.	One	1.937572
Sept. 28	3:00 p.m.	One	1.037561
Oct. 25	4:30 p.m.	Three	1.937556

**Table 2:** Electromotive force of NBS standard cell No. 1064 during a coulometric run (May 22, 1958)

Time	emf	Time	emf
			
	***v***		***v***
8:45 a.m.	1.0182103	12:10 p.m.	1.0182103
8:47 a.m.	1.0182103	12:18 p.m.	1.0182102
8:50 a.m.	1.0182103	12:51 p.m.	1.0182102
9:20 a.m.	start	12:54 p.m.	1.0182102
9:36 a.m.	1.0182103	1:26 p.m.	1.0182102
9:39 a.m.	1.0182103	1:29 p.m.	1.0182103
10:11 a.m.	1.0182103	2:06 p.m.	1.0182103
10:14 a.m.	1.0182103	2:09 p.m.	1.0182103
10:51 a.m.	1.0182103	2:20 p.m.	end
10:54 a.m.	1.0182103	2:28 p.m.	1.0182103
11:28 a.m.	1.0182103	2:29 p.m.	1.0182103
11:31 a.m.	1.0182103	……………	……………
	
Mean value			1.0182103

**Table 3:** Measurements of the *IR* drop by circuit No. 2 figure 5^a^

Time	*IR* setting^b^	Swings to left	Swings to right	Correction	Correct *IR* drop
					
	*v*	*cm*	*cm*	*μv*	*v*
9:20 a.m.	start	………………	………………	…………	…………
9:37 a.m.	1.0182103	0.7, 0.6, 1.1	0.3, 0.6, 1.1	−0.3	1.0182100
10:12 a.m.	1.0182103	.3, .3, 0.2	.2, .4, 0.2	.0	1.0182103
10:52 a.m.	1.0182103	1.0, .1, .7	1.2, .2, .4	.0	1.0182103
11:29 a.m.	1.0182103	0.5, .2, .6	0.2, .7, .4	.0	1.0182103
12:11 p.m.	1.0182103	.2, .1, .3	.2, .1, .3	.0	1.0182103
12:16 p.m.	1.0182103	.5, .5, .2	.3, .5, .3	−.1	1.0182102
12:52 p.m.	1.0182102	.7, .6, .4	.7, .6, .4	.0	1.0182102
1:27 p.m.	1.0182102	.4, .2, .5	.4, .3, .6	.1	1.0182103
2:07 p.m.	1.0182103	1.3, .1, .2	1.3, .1, .2	.0	1.0182103
2:20 p.m.	end	………………	………………	…………	…………
	
Mean value					1.0182103

a On May 22, 1958.

b As given by circuit No. 1, figure 5.

**Table 4:** Constants of the equation for the resistance of the standard resistors as a function of temperature

$R_{t}=\left(nv+k\right)\;\left[1+\alpha \left(t-25{}^{\circ}C\right)+\beta {\left(t-25{}^{\circ}C\right)}^{2}\right]$						

Resistor No. (NBS)	Nominal value (*nv*)	*k*			*α*×10^6^	*β*×10^6^
		March 1956	April 1957	April 1958		
						
	*Ohms*	*Ohm*	*Ohm*	*Ohm*		
204	10	+0.00002	0	+0.00001	+5.8	−0.53
205	10	+.00002	0	−.00001	+6.0	−.56
12	10	−.00044	−0.00046	−.00047	+3.4	0
17	10	+.00078	+.00078	+.00077	−1.0	−.24
8	1	+.000530	+.000529	+.000528	+9.2	−.50
12	1	0	−.000009	−.000016	−3.8	−.12
13	1	+.000017	+.000617	+.000617	+5.4	−.51

**Table 5:** Load resistances of standard resistors

Current	Resistor No. (NBS)	Temperature	Total resistance (no load correction)	Total resistance (with load correction)
				
*amp*		*°C*	*Ohms*	*Ohms*
0.1	204	25.1	10.000026	10.000026
.2	204 205	24.8 24.8	9.999988 9.999998(a)	
			4.999997	4.999997
.3	12 13 8	25.2 25.2 25.2	$\begin{array}{c} 0.999992\\ 1.000620\\ 1.000527 \end{array}\}$ (b)	
			3.001139	3.001139
.5	12 13	25.4 25.3	0.999991 1.000621(b)	
			2.000612	2.0006104

a =in parallel;

b =in series.

**Table 6:** Magnitude of the current in run of May 22, 1958

Time	*IR* drop	Temperature resistor 12	Resistance of resistor 12	Temperature resistor 17	Resistance of resistor 17	Total resistance	Current
							
	*v*	*°C*	*Ohms*	*°C*	*Ohms*	*Ohms*	*amp*
9:20^a^	1.018210_3_	24.5	9.9995130	24.5	10.0007744	5.000072	0.20303913
9:37	1.018210_0_	24.6	9.9995164	24.6	10.0007736	5.000073	.20363903
10:12	1.018210_3_	25.1	9.9995334	25.1	10.0007690	5.000076	.20363896
10:52	1.018210_3_	25.4	9.9995436	25.4	10.0007650	5.000077	.20363892
11:29	1.018210_3_	25.6	9.9995504	25.6	10.0007631	5.000078	.20363888
12:11	1.018210_3_	25.7	9.9995538	25.7	10.0007618	5.000079	.20363884
12:16	1.018210_2_	25.8	9.9995572	25.8	10.0007605	5.000079	.20363882
12:52	1.018210_2_	25.9	9.9995606	25.9	10.0007591	5.000080	.20363878
1:27	1.018210_3_	26.0	9.9995640	26.0	10.0007570	5.000080	.20363880
2:07	1.018210_3_	26.1	9.9995674	26.1	10.0007561	5.000081	.20363876
2:20^b^	1.018210_3_	26.1	9.9995674	26.1	10.0007561	5.000081	.20363876
		
Mean values						5.000078	0.20363887

a Start,

b End.

**Table 7:** Spectrochemical analyses of the various silver samples

Element	Mint sample No. 1	Mint sample No. 2	Anode 1	Anode 2	Anode 4	Anode 5
						
	*ppm*	*ppm*	*ppm*	*ppm*	*ppm*	*ppm*
Al	0.5	0.05	0.00_5_	0.00_5_	0.01	0.02_5_
Ca	.5	b.25_5_	.01	.01_5_	.01	.00_5_
Cu	a4.0	4.0	.2_5_	.2_5_	.10	.07_5_
Fe	0.5	0.1_5_	.1	.1_5_	.05	.1
Mg	.20	.02	.01	.01_5_	.01	.01
Mn	.5	……….	……….	……….	……….	……….
Pb	……….	……….	……….	……….	……….	……….
Si	1.0	.10	……….	.10	……….	.57
Sn	0.2	……….	……….	……….	0.05	……….
Pd	.5	……….	……….	……….	……….	……….
Au	.5	……….	……….	……….	……….	……….
						
Total	8.40	4.57_5_	.37_5_	.53_5_	.23	.78_5_

a Photometric method gave 3.5 ppm.

b Subfigures refer to differences between top and bottom of samples; differences, as seen, are insignificant.

**Table 8:** Decomposition pressure of silver oxide, *Ag*_2_*O*

Temperature	Pressure of oxygen	Reference
		
*°C*	*atm*	
173	0.555	[30]
178	.670	[30]
183	.796	[30]
188	.943	[30]
191	1.039	[30]
302	20.5	[28]
325	32.0	[28]
374	74.3	[29]
403	114.5	[29]
445	207.0	[28]
452	213.5	[29]
467	257.8	[29]
484	323.5	[29]
500	388.3	[29]

**Table 9:** Magnitudes (nominal) of current and resistance used in the faraday determinations

Measurements	Nominal current	Nominal resistance	Resistors^a^	Resistor code
				
	*amp*	*Ohms*		
1	0.09	11	13(1Ω), 12(10Ω), *S*	A
2	.10	10	204(10Ω)	B
2	.15	6⅔	204(10Ω), 17(10Ω) *S*, 12(10Ω) *P*	C
1	.17	6	12(10Ω), 17(10Ω)*P*, 13(1Ω) *S*	D
2	.20	5	204(10Ω), 205(10Ω), *P*	E
12	.20	5	12(10Ω), 17(10Ω), *P*	F
26	.33	3	8(1Ω), 12(1Ω), 13(1Ω) *S*	G
7	.50	2	12(1Ω), 13(1Ω) *S*	H

a *S*=series, *P*=parallel.

**Table 10:** Final results of the electrochemical equivalent of silver

Experiment No.	Date	Time	*IR* drop	Resistor code	Resistance	Current	Coulombs^a^	*E_r_*	Silver corroded, Ag_m_	Sediment	Electrochemical equivalent	
											Uncorrected	Corrected

Mint silver No. 1—Hydrogen, 2 hr, 862° C, Helium (118 hr), not melted, *o*^c^												

		*sec*	*v*		*Ohms*	*amp*	*amp-sec*	*v*	*g*	*g*	*mg coulomb*^−1^	*mg coulomb*^−1^
1	11-28-56	7,199.959	1.0182138	H	2.000609	0.5089519	3,664.4323	0.125	4.096763	0.020678	1.117980	d1.117971 (17)
2	12-13-56	18,000.013	1.0182133	E	5.000001	.2036426	3, 665.5690	.091	4.097912	.051298	1.117947	1.117938 (17)
3	12-18-56	10,800.053	1.0182139	G	3.001136	.3392761	3, 664.1979	.201	4.096574	.767097	1.118000	1.117991 (17)

Mint silver No. 1—Hydrogen, 96 hr, 860° C, Helium (250 hr), not melted, *e*^c^												

4	4-11-57	10,802.313	1.0182129	G	3.001130	0.3392765	3, 664.9702	0.122	4.097160	0.032410	1.117925	1.117949 (16)
5	4-16-57	10,200.067	1.0182102	G	3.001126	.3392761	3, 460.6383	.124	3.868779	.058122	1.117938	1.117962 (16)
6	4-18-57	9,900.074	1.0182118	G	3.001125	.3392767	3, 358.8637	.117	3.754894	.056315	1.117906	1.117930 (16)

Mint silver No. 2—Vacuum, 1 hr, 550° C, not melted, *o*												

7	3-6-57	13,199.981	1.0182131	G	3.001133	0.3392762	4, 478.4388	0.111	5.006858	0.471592	1.117992	1.117986 (12)

Mint silver No. 2—Vacuum, 5 hr, 550° C, not melted, *o*												

8	3-11-57	9,900.062	1.0182101	G	3.001130	0.3392756	3,358.8491	0.098	3.755131	0.102182	1.117982	1.117975 (12)
9	3-14-57	10, 500.078	1.0182125	G	3.001139	.3392754	3,562.4159	.159	3.982688	.104516	1.117974	1.117968 (12)
10	4-8-57	10,800.037	1.0182131	G	3.001137	.3392758	3, 664.1903	.112	4.096469	.118233	1.117974	1.117968 (12)

Mint silver No. 2—Vacuum, 10 hr, 550° C, not melted, (*e*′)^c^												

11	4-30-57	10,800.040	1.0182131	G	3.001143	0.3392751	3,664.1842	0.100	4.096415	0.075844	1.117961	1.117970 (4)
12	5-2-57	10.799.967	1.0182133	G	3.001141	.3392754	3,664.1627	.088	4.096383	.090773	1.117959	1.117968 (4)
13	5-8-57	10,800.117	1.0182139	G	3.001143	.3392754	3,664.2125	.144	4.096498	.065560	1.117975	1.117984 (4)

Mint silver No. 2—Hydrogen, 96 hr, 860° C, not melted, *e*												

14	5-28-57	10,500.032	1.0182132	G	3.001141	0.3392754	3,562.4021	0.104	3.982489	0.228977	1.117922	1.117936 (8)
15	6-3-57	10,800.083	1.0182129	G	3.001137	.3392757	3,664.2051	.106	4.096410	.244528	1.117953	1.117967 (8)

Mint silver No. 2—Hydrogen, 101 hr, 860° C, not melted, *e*												

16	6-26-57	10,200.407	1.0182123	G	3.001139	0.3392753	3,460.7454	0.110	3.869000	0.208168	1.117967	1.117980 (7)
17	7-2-57	10,500.086	1.0182129	G	3.001137	.3392757	3,562.4233	.105	3.982423	.269498	1.117897	1.117910 (8)

Anode 1—Vacuum, 0.5 hr, 975° C, melted, *e*												

18	1-21-58	18,000.021	1.0182119	F	5.000071	0.2036395	3,665.5447	0.106	4.098127	0.016516	1.118013	1.118014 (1)
19	1-28-58	21,600.031	1.0182112	C	6.666528	.1527349	3,299.0730	.104	3.688228	.020922	1.117959	1.117960 (1)
20	2-12-58	23,400.024	1.0182101	A	11.000122	.0925635	2,165.9930	.106	2.421491	.392851	1.117959	1.117960 (1)

Anode 2—Vacuum, 1 hr, 975° C, melted, *e*												

21	2-26-58	18,000.048	b1.0181863	F	5.000072	0.2036343	3,665.4270	0.089	4.097824	0.017288	1.117966	1.117968 (1)
22	3-4-58	18,000.036	1.0182090	F	5.000075	.2036388	3,665.5053	.100	4.097795	.007993	1.117934	1.117936 (1)
23	3-11-58	18,000.075	1.0182098	F	5.000073	.2036390	3,665.5169	.105	4.097900	.005706	1.117960	1.117961 (1)
24	3-18-58	21,600.055	1.0182098	D	6.000684	.1696823	3,665.1505	.107	4.097576	.002229	1.117983	1.117984 (1)
25	5-28-58	18,000.018	1.0182104	F	5.000075	.2036390	3,665.5048	.149	4.097921	.066674	1.117969	1.117970 (1)

Anodes 1 and 2 combined—0.75 hr, 965° C, melted, *e*												

26	7-9-58	15,300.060	1.0182111	C	6.666547	0.1527344	2,336.8449	0.120	2.612583	0.002269	1.117996	1.117997 (1)

Anode 4—Hydrogen, 21 hr, vacuum, 975° C, melted, *e*												

27	5-20-58	18,000.015	1.0182100	F	5.000075	0.2036390	3,665.4555	0.127	4.097800	0.000613	1.117951	1.117951 (0)
28	5-22-58	18,000.016	1.0182103	F	5.000078	.2036389	3,665.5027	.134	4.097844	.000631	1.117949	1.117949 (0)

Anode 5—Hydrogen, 7.75 hr, vacuum, 975° C, melted, *e*												

29	6-18-58	17,999.998	1.0182105	F	5.000077	0.2036390	3,665.4962	0.109	4.097935	0.000940	1.117976	1.117976 (1)
30	6-24-58	17,999.945	1.0182105	F	5.000076	.2036390	3,665.4904	.115	4.097861	.000796	1.117957	1.117958 (1)
31	7-2-58	17,999.955	1.0182110	F	5.000079	.2036390	3,665.4922	.132	4.097897	.000636	1.117966	1.117967 (1)

a Includes corrections for any initial off-balance and coulombs utilized in side-circuit measurements of reference silver electrode.

b NBS cell No. 1063 used instead of NBS cell No. 1064.

c *o*-impurities are metallic oxides, *e*-impurities are metallic, *e*′-impurities are metallic oxides except Fe and Cu which are metallic.

d Uncertainties in purity corrections, given in parentheses.

**Table 11:** Distinct groups of values for the electrochemical equivalent of silver

Group No.	Silver	Number of runs	Treatment	Hours of treatment	Electrochemical equivalent of silver (average)	Group average for electrochemical equivalent of silver	$S_{\bar{x}}\times 10^{6}$
							
					*mg coulomb*^−1^	*mg coulomb*^−1^	
1	$\left\{ \begin{array}{l} \text{Mint No}.\;1\\ \text{Mint No}.\;1 \end{array}\right.$	3 3	Hydrogen Hydrogen	2 96	1.117967 1.117947	} 1.117957	8.6
2	$\left\{ \begin{array}{l} \text{Mint No}.\;2\\ \text{Mint No}.\;2 \end{array}\right.$	2 2	Hydrogen Hydrogen	96 101	1.117952 1.117945	} 1.117948	10.6
3	$\left\{ \begin{array}{l} \text{Purified}\\ \text{Purified} \end{array}\right.$	3 2	Hydrogen Hydrogen	7¾ 21	1.117967 1.117950	} 1.117960	9.5
	Average	15	Hydrogen	……………	1.117956	1.117956	5.5
4	$\left\{ \begin{array}{l} \text{Mint No}.\;2\\ \text{Mint No}.\;2\\ \text{Mint No}.\;2 \end{array}\right.$	1 3 3	Vacuum Vacuum Vacuum	1 5 10	1.117986 1.117970 1.117974	} 1.117974	8.0
5	$\left\{ \begin{array}{l} \text{Purified}\\ \text{Purified}\\ \text{Purified} \end{array}\right.$	3 1 5	Vacuum Vacuum Vacuum	½ ¾ 1	1.117978 1.117997 1.117960	} 1.117972	7.0
	Average	16	Vacuum	……………	1.117973	1.117973	5.3
						
	Total average	31	……………	……………	1.117965	1.117965	3.8

**Table 12:** Final values for the electrochemical equivalent of silver

Sample No.	Mint silver	Δ	Sample No.	Purified silver	Δ
					
		*ppm*			*ppm*
1	1.117972	0	18	1.118014	+4.2
2	1.117939	−3.3	19	1.117960	−1.2
3	1.117992	+2.0	20	1.117960	−1.2
4	1.117970	−0.2	21	1.117968	−0.4
5	1.117983	+1.1			
			22	1.117936	−3.6
6	1.117951	−2.1	23	1.117961	−1.1
7	1.117986	+1.4	24	1.117984	+1.2
8	1.117975	+0.3	25	1.117970	−0.2
9	1.117968	−0.4	26	1.117997	+2.5
10	1.117968	−.4			
			27	1.117978	+0.6
11	1.117970	−.2	28	1.117976	+.4
12	1.117968	−.4	29	1.117986	+1.4
13	1.117984	+1.2	30	1.117968	−0.4
14	1.117957	−1.5	31	1.117977	−.5
15	1.117988	+1.6			
16	1.118001	+2.9			
17	1.117931	−4.1			
	
Average					1.117972
$S_{\bar{x}}$					5.9×10^−6^

Uncertainty (95% confidence limit for the mean) = 2.093

$S_{\bar{x}}=\pm 0.000012$

**Table 13:** Isotopic composition of isotopieally enriched silvers

	Ag^107^	A^109^	Notes
			
Silver “107”	$\{\;\begin{array}{l} 98.81\\ 98.804\\ 98.79_{8} \end{array}$	1.19 1.196 1.20_2_	Oak Ridge. NBS. NBS, bias corrected.
Silver “109”	$\{\;\begin{array}{l} 0.94\\ .915\\ .91_{0} \end{array}$	99.06 99.085 99.09_0_	Oak Ridge. NBS. NBS, bias corrected.

**Table 14:** Isotope ratios in synthetic blends of silvers

Experiment No.	Weight of silver “107”	Weight of silver “109”	Isotope ratio		Bias ratio (calculated/measured)
			Calculated	Measured	
					
	*g*	*g*			
1	0.107004	0.100151	1.07998	b1.08628 (8)	0.99417
2	.107135	.101350	1.06873	1.07462 (8)	.99452
3	.108413	.101766	1.07688	1.08118 (8)	.99602
4^a^	.107736	.101732	1.07065	1.07599 (6)	.99504
5^a^	.110689	.103758	1.07835	1.08555 (6)	.99337
	
Weighted average					0.99467
	
	Isotope ratio	Bias ratio	Bias × 1.08148	Atomic weight	
				Physical	Chemical
1	1.07970	0.993942	1.07493	107.9030	107.8734
2	1.06848	.994283	1.07530	107.9028	107.8732
3	1.07663	.995790	1.07693	107.9021	107.8724
4^a^	1.07040	.994803	1.07586	107.9026	107.8729
5^a^	1.07810	.993136	1.07406	107.9034	107.8738
				
Weighted average		.99444	1.07547	107.9028	107.8731

a Electrolytically purified.

b Numbers in parentheses equals number of analyses.

**Table 15:** Results with silver containing oxygen

Experiment No.	Date	Time	IR drop	Resistor code	Resistance	Current	Coulombs^a^	*E_r_*	Silver corroded, Ag*_m_*	Sediment	Electrochemical equivalent		
											Uncorrected	Corrected^b^	Corrected^c^

Mint silver No. 2—no treatment, *O*^d^													

		*sec*	*v*		*Ohms*	*amp*	*amp-sec*	*v*	*g*	*g*	*mg coulomb*^−1^	*mg coulomb*^−1^	*mg coulomb*^−1^
A1–1….	1–3–57	10,799.982	1.0182141	G	3.001127	0.3392772	3,664.1873	0.094	4.100057	0.043981	1.119118	1.119111	1.118776
A1–2….	1–8–57	10,799.945	1.0182132	G	3.001129	.3392767	3,664.1693	.099	4.100600	.059223	1.119108	1.119101	1.118766

Mint silver No. 2—etched only, *o*^d^													

A1–3….	2–28–57	10,800.040	1.0182132	G	3.001135	0.3392760	3,664.1940	0.091	4.100969	0.033223	1.119201	1.119195	1.118859

Mint silver No. 2—annealed only, *o*^d^													

A1–4….	1–17–57	9,000.244	1.0182127	G	3.001127	0.3392768	3,053.5735	0.117	3.414747	0.106558	1.118279	1.118272	1.118110
A1–5….	1–23–57	9,600.043	1.0182119	G	3.001133	.3392758	3,257.0620	.097	3.642077	.101450	1.118209	1.118202	1.118040

Mint silver No. 2—annealed and etched, *o*^d^													

A1–6….	2–11–57	8.999.949	1.0182127	G	3.001131	0.3392763	3,053.4690	0.105	3.414218	0.121079	1.118144	1.118138	^*^1.117975
A1–7….	2–18–57	9,600.009	1.0182125	G	3.001133	.3392760	3,257.0522	.099	3.641862	.162788	1.118147	1.118140	^*^1.117977

Mint silver No. 1—annealed and etched, *o*^d^													

A1–8….	7–17–56	10,800.140	1.0182203	H	2.000612	0.5089544	5,496.7786	0.082	6.146287	0.697158	1.118162	1.118152	^*^1.117988
A1–9….	8–3–56	7,200.042	1.0182135	H	2.000612	.5089510	3,664.4684	.081	4.097390	.508128	1.118140	1.118131	^*^1.117968
A1–10….	8–9–56	7,200.107	1.0182119	H	2.000612	.5089502	3,664.4956	.093	4.097430	.296137	1.118143	1.118133	^*^1.117971
A1–11….	8–15–56	11,999.980	1.0182153	G	3.001145	.3392756	4,071.3004	.087	4.552775	.484088	1.118261	1.118251	^*^1.118088

a Includes corrections for any initial off-balance and coulombs utilized in side-circuit measurements of reference silver electrode.  Average (total)…. 1.118020

b With corrections for metallic impurities.    Average (^*^)…. 1.117995

c With corrections for metallic impurities and estimated corrections for oxygen solubility.        Average (^*^omitting A1–11)omitting 1.117976

d Impurities are metallic oxides.

**Table 16:** Solubility of oxygen in silver

t° C	Pressure (mm)					
	50	100	(160)	200	400	800

Volume O_2_ (NTP) per volume Ag						

200	0.030	0.050	(0.064)	0.071	0.100	0.142
300	.021	.032	(.040)	.045	.070	.097
400	.020	.031	(.039)	.044	.061	.087
500	.022	.034	(.043)	.048	.067	.095
600	.033	.047	(.059)	.066	.093	.132
700	.048	.068	(.087)	.096	.134	.193
800	.088	.124	(.157)	.175	.247	.354

**Table 17:** Results of specific effects of hydrogen on silver

Experiment No.	Date	Time	*IR* drop	Resistor code	Resistance	Current	Coulombs^a^	*E_r_*	Silver corroded, Ag*_m_*	Sediment	Electrochemical equivalent		
											Uncorrected	Corrected^b^	Corrected^c^

Mint silver No. 1—excess hydrogen, not melted, *e*^d^													

		*sec*	*v*		*Ohms*	*amp*	*amp-sec*	*v*	*g*	*g*	*mg coulomb*^−1^	*mg coulomb*^−1^	*mg coulomb*^−1^
A2–1	8–28–56	14,400.096	1.0182142	G	3.001143	0.3392755	4,885.5993	0.092	5.461195	0.065717	1.117815	1.117839	1.117888
A2–2	8–30–56	10,800.455	1.0182125	H	2.000613	.5089503	5,496.8942	.126	6.144322	.114476	1.117781	1.117805	1.117854
A2–3	9–17–56	8,400.100	1.0182130	H	2.000613	.5089505	4,275.2345	.119	4.778785	.078265	1.117783	1.117808	1.117857
A2–4	9–19–56	10,800.311	1.0182140	G	3.001142	.3392755	3,664.2805	.097	4.095865	.035293	1.117782	1.117806	1.117855
A2–5	9–24–56	22,500.065	1.0182133	B	10.000017	.10182115	2,290.9822	.067	2.560846	.036912	1.117794	1.117818	1.117868
A2–6	9–27–56	22.500.075	1.0182139	B	9.999983	.10182156	2.200.9923	.073	2.560977	.068333	1.117846	1.117871	1.117920
A2–7	10–16–56	7,203.775	1.0182142	H	2.000012	.5089513	3,006.3703	.110	4.098252	.052391	1.117795	1.117820	1.117869
A2–8	10–18–56	10,799.954	1.0182138	G	3.001142	.3392754	3,664,1583	.098	4.095960	.074096	1.117845	1.117869	1.117918
A2–9	10–23–56	18,000.038	1.0182138	E	5.000001	.2036427	3,665.5759	.092	4.097376	.056630	1.117799	1.117823	1.117872
												
												Average…..1.117878	

Anode 3—melted in hydrogen													

A2–10	4–8–58	18,000.019	1.0182100	E	5.000085	0.2036385	3,665.4965	0.101	4.098482	0.003117	1.118125	……….	……….
A2–11	4–28–58	17,999.998	1,0182097	E	5.000087	.2036384	3,665.4903	.126	4.098288	.000875	1.118074	……….	……….

a Includes correetions for any initial off-balance and coulombs utilized in side-circuit measurements of reference silver electrode.

b With corrections for metallic impurities.

c With corrections for metallic impurities and estimate corrections for hydrogen solubility.

d Impurities are metallic.

**Table 18:** Solubility of hydrogen in silver

t° C	Pressure (mm)				
	50	100	200	400	800

Volume H_2_ (NTP) per volume silver					

200	0	0	0	0	0
300	Trace	Trace	Trace	Trace	Trace
400	Trace	0.002	0.003	0.004	0.006
500	0.003	.004	.006	.008	.012
600	.005	.007	.010	.014	.019
700	.006	.009	.013	.018	.025
800	.009	.013	.018	.025	.036
900	.012	.016	.023	.033	.046

**Table 19:** Spectrochemical analyses of anode No. 3

Element	Top^a^	Bottom
		
	*ppm*	*ppm*
A1	<1.0	<1.0
Cd	0.01	0.01
Cu	0.1–0.2	0.1–5.0
Fe	0.05–1.0	0.2–5.0
Mg	0.1–0.5	0.01–2.0
Ni	………..	<10
Pb	………..	<50
Si	<1	0.1–0.5
Sn	………..	<5

a Top of anode near supporting wires.

## Footnotes

1 Figures in brackets indicate the literature references at the end of this paper.

2 The iodine coulometer had been studied earlier by Herroun [4], Danneel [5], Kreider [6], and Gallo [7] but these experimenters did not claim high accuracy for their work.

3 In recent years two new methods, one a physical method using an omegatron [10] and the other an electrochemical method involving the anodic oxidization of sodium oxalate [11] have been reported. These are not considered here.

4 Spectrochemical analysis gave different results for etched and unetched mint silver; the unetched silver contained a somewhat higher percentage of impurities. Photometric chemical analysis confirmed this finding and showed that unetched silver contained an appreciable amount of iron (4 ppm) whereas etched silver showed an insignificant amount of iron (the iron apparently came from the steel rolls used in forming the sheets of silver). Undoubtedly the larger amount of impurities found in unetched silver arose from surface Impurities. Only the results obtained with etched silver were used in the final evaluation of the faraday.

5 Ag_precipitated_ and Σ′ are actually given by:

$${\text{Ag}}_{\text{precipitated}}=X\left({\text{Ag}}_{m}+{\text{Ag}}_{\text{precipitated}}+{\text{SiO}}_{2}+\Sigma i_{\text{inert}}\right)$$

and

$$\Sigma \prime i=Y\left({\text{Ag}}_{m}+{\text{Ag}}_{\text{precipitated}}+{\text{SiO}}_{2}+\Sigma i_{\text{inert}}\right),$$

wherein a series of successive approximations are required to obtain Ag_precipitated_ and Σ′*i*. However, the amounts of impurities were so small that Ag*_m_* may be used for the quantities In parentheses in calculating the faraday. Differences in the faraday obtained by using Ag*_m_* for the quantities in parentheses are less than 1 ppm.

6 Computed in each case from $S_{\bar{x}}=s/\sqrt{n}$ where *n* is the number of values entering into the mean and *s* is the estimate of the standard deviation of a single value obtained by pooling the dispersions within the 12 groups given in table 10, and is based on 19 degrees of freedom.

7 The value of | difference of means |/(standard deviation of difference) = 2.37 exceeds 2.093, the critical value (0.05 probability point) of Student’s *t* for 19 degrees of freedom.

8 This uncertainty includes allowances of 0.0007 in the atomic weight of silver and 0.000007 in the electrochemical equivalent as the possible effect of systematic errors and 2.1 times the standard deviation in the ratio of the atomic weight to the electrochemical equivalent of silver as the limit to the effect of random errors.

9 These values are based on the electrical standards as presently maintained at the National Bureau of Standards. Recently, Driscoll [37] by means of an electrodynamometer of the Pellat type found that 1 NBS amp=1.000013±0.000008 abs amp. In the same year Driscoll and Cutkosky [38] by using a current balance found that 1 NBS amp=1.000008±0.000006 abs amp. Since the acceleration of gravity is a common factor in these two determinations, it was taken out before averaging, and then reentered after averaging. On doing this Driscoll and Cutkosky [38] obtained 1 NBS amp = 1.000010 ±0.000005 abs amp. On this basis the values of the faraday given above would be 10 ppm higher. However, since the electrical standards as maintained are used in the dissemination of the units and in international comparisons, the values of the faraday based on the electrical standards as maintained are the ones given.

## 12. Appendix 1. Effect of Oxygen

In this paper, it was stressed that efforts were made to remove oxygen and silver oxide from the starting silver. To show specifically the effect of oxygen three runs were made in which no attempt was made to remove the oxygen as it may normally exist in silver, and eight runs were made with silver in which only a superficial attempt was made to remove the oxygen, i.e., it was simply heated to dull redness in a Bunsen flame.

All measurements, electrical and chemical, were made with the same care and precision as in the best measurements. The treatments given to the silver were as follows:

*Experiments A1–1 and A1–2:* Mint silver No. 2 as received from the U.S. Mint at Philadelphia, Pa., was given no treatment whatsoever.
*Experiment A1–3:* Mint silver No. 2 was slightly etched in 10-percent aqueous ammonium hydroxide; given no other treatment.
*Experiments A1–4 and A1–5:* Mint silver No. 2 was heated to dull redness.
*Experiments A1–6 and A1–7:* Mint silver No. 2 was heated to dull redness and etched slightly in 10-percent aqueous ammonium hydroxide.
*Experiments A1–8, A1–9, A1–10, and A1–11:* Mint silver No. 1 was treated in the same manner as samples A1–6 and A1–7.

The results obtained with these experiments are given in table 15. Inspection of table 15 shows that the values for the electrochemical equivalent are high, especially for those samples not heated to dull redness. Mere heating to dull redness decomposes at least part of the silver oxide and removes part but not all of the dissolved oxygen. That these high results are a consequence of dissolved oxygen may be shown by the following calculations. The data of Steacie and Johnson [26] on the solubility of oxygen in silver at various temperatures and pressures are reproduced in table 16.

The solubility of oxygen in silver passes through a minimum at about 400° C. Steacie and Johnson concluded that oxygen exists as the element in silver above 400° C and as the lower oxide, Ag_2_O, below 400° C. Since normal atmospheric air contains about 21 percent of oxygen the partial pressure of oxygen in air at normal temperature and pressure is 160 mm. The solubility of oxygen in silver at this pressure was obtained by interpolation of the data of table 16 and the interpolated values are given in parentheses in column 4 of table 16. Unfortunately, Steacie and Johnson did not extend their measurements below 200° C. They stated, however, that “it appears probable that the solubility is considerably greater at room temperature than at 200° C”. To obtain an *estimate* of the solubility at 25° C the data at 160 mm pressure were fitted to the distorted parabolic equation:

$$-\log\;S=-\log\;S_{m}+a{\left(t-m\right)}^{2}+ct^{2}$$

where *S_m_* is the minimum solubility (*S*); *m*, the temperature (400° C) for the minimum solubility; *t*, deg Celsius; and *a, b*, and *c* are empirical constants. Values of log *S_m_, a, b*, and *c* were determined by the method of least squares; then the equation with numerical values is:

$$-\log\;S=1.4089-4.0\times 10^{-6}{\left(t-400\right)}^{2}+2.4\times 10^{-8}t^{2}.$$

For 25° C this equation gives 0.15 for the volume of oxygen dissolved in a unit volume of silver at 25° C and 160 mm of oxygen pressure. Now taking the density of silver as 10.50 the volume of oxygen associated with 1 g of silver is 0.0143 ml. At 25° C, this oxygen exists combined with silver in the form of the lower oxide, Ag_2_O. Thus, 0.0003 g of Ag_2_O is associated with 1 g of silver or silver contains 0.03 percent of Ag_2_O. This Ag_2_O will be chemically soluble in perchloric acid, and thus if corrections are not made for its presence high values (as seen in column 13 of table 15) for the electrochemical equivalent of silver will result. Values obtained when the above estimated corrections are made are given in the last column of table 15 (first 3 values).

When the silver is heated to dull redness in air the situation is quite different. According to Bone and Wheeler [39] the temperature of silver at dull redness is about 650° C. From table 16 the volume of oxygen associated with 1 volume of silver at 650° C and 160-mm pressure is 0.074; this is equivalent to 0.00001006 g oxygen/g of silver. Now when the silver is cooled to room temperature (25° C) this oxygen will exist combined with silver as Ag_2_O or 0.0001457 g of Ag_2_O is associated with 1 g of silver. Since this Ag_2_O will be chemically soluble in perchloric acid, high values (see column 13 of table 15) of the electrochemical equivalent of silver will result if corrections are not made for the presence of Ag_2_O. Values obtained when these estimated corrections are made for experiments A1–4 to A1–11, inclusive, are given in the last column of table 15. In this case, corrections can be made without an extrapolation, or use of an empirical equation, since the amount of Ag_2_O can be calculated from the amount of oxygen dissolved in silver at 650° C and for which data are available (table 16).

The average value for the electrochemical equivalent of unannealed silver is 1.118800 mg coulomb^−1^; if only the annealed and etched samples are used the average is 1.117995 (omitting the last value the average is 1.117976). These average values for the annealed samples agree remarkably well with the final value given in the text; the agreement is remarkable in view of the rough estimates that were made of the oxygen content. For the unannealed samples the values are still high indicating that underestimates of the amount of Ag_2_O in silver result from the use of the above parabolic equation to calculate oxygen content and from that the Ag_2_O content. One would not pretend to use this procedure to arrive at an accurate and precise value of the faraday; this appendix was included here to show the need for the removal of oxygen or silver oxide from the starting silver.

## 13. Appendix 2. Effect of Hydrogen

It was stressed earlier that all hydrogen must be removed from the silver. During the course of these investigations two specific effects of hydrogen were noted. Mint silver No. 1 was used in a series of nine experiments in which the silver was treated in a silica tube with hydrogen for 72 hr at 875.5° C and then cooled abruptly in helium to room temperature within 2 hr. Nine coulometric measurements were then made with this treated silver with the same care and precision, as were made with the best measurements, and the results are given in table 17. These results became puzzling in view of later experiments in that the values for the electrochemical equivalent of silver were quite low. The presence of hydrogen would lower the electrochemical equivalent but when mint silver No. 2 was treated in like manner the final results were quite different and agreed closely with the vacuum values. The reason for this difference was not at once apparent until it was found that mint silver No. 1 contained traces of palladium and gold which were not detected in mint silver No. 2. Palladium could catalyze the reaction Ag^+^+(½)H_2_→Ag+H^+^ in a manner similar to that at a hydrogen electrode.

Steacie and Johnson [40] in a comprehensive study of the solubility of hydrogen obtained the results given in table 18. Since the silver in the present experiments was abruptly cooled from 875.5° C to room temperature in 2 hrs and with only superficial replacement of hydrogen by helium it is assumed that the dissolved hydrogen was not completely displaced or removed. Furthermore, it is known that metal-hydrogen systems show marked hysteresis in their isotherms [41]. Thus, 0.046 volume of hydrogen per volume of silver was used since the silver was heated at 875.5° C at a pressure slightly exceeding atmospheric. Thus 0.000000394 g hydrogen is associated with 1 g of silver and this amount of hydrogen will produce 0.0000425 g of silver by reduction of Ag^+^ ion. When corrections are made for the Ag^+^ ions which are reduced the values given in the last column of table 17 are obtained. The average value, after these corrections, for the electrochemical equivalent of silver is 1.117878 mg coulomb^−1^. This value differs by 8 parts in 100,000 from the value given in the main part of this paper and is in the direction expected for silver containing hydrogen in excess of that given by the solubility data of Steacie and Johnson.

Steacie and Johnson worked with silver foil and stated that there was no adsorption of hydrogen and that only absorption or solution of hydrogen occurred in silver. Bone and Wheeler [39], however, found that silver gauze which had been repeatedly used, i.e., had a roughened surface retained as much as 1.3 volumes of hydrogen per unit volume of silver. Also it is known that Pd-Ag alloys absorb more hydrogen than pure silver [42]. Accordingly, the data in table 18 may be considered as minimum solubility values and that a small additional amount of hydrogen was probably adsorbed on the surface of the silver, which would lead to a further but small rise in the corrected values of the electrochemical equivalent of silver given in table 17.

Another experiment involved the melting of purified silver (anode No. 3) in an atmosphere of hydrogen. The anode was first heated in *vacuo* from room temperature to 530° C in 17 hr, hydrogen was then introduced and the temperature raised to 975° C in 4 hr. The silver in the molten state was kept under hydrogen for 15 min. The hydrogen was then replaced over a 45-min period with helium. The tube was then evacuated, cooled slowly to 900° C (60° C below melting point), and then cooled abruptly to room temperature. Melting the silver in hydrogen caused blistering of the silver and when the anode was electrolytically corroded in the first experiment it was seen that the anode contained cavities. These cavities could have caused not only small uncertainties in the vacuum corrections but also could have prevented the complete removal of electrolyte from the anode when it was washed with conductivity water in the usual manner. Furthermore, the spectrochemical analyses of the silver (anode No. 3) given in table 19 indicate that the silver extracted considerable amounts of impurities from the silica tube. This observation is similar to that found by Baxter and Lundstedt [35] who observed that when silver is melted on pure lime in dry hydrogen that the silver became contaminated with calcium. Owing to the relatively large contamination and the cavities in the silver meaningful corrections for impurities could not be made in these experiments; only the uncorrected values for the electrochemical equivalent are given in the last two rows of table 17.

This appendix shows the effects of hydrogen on determining the electrochemical equivalent of silver and emphasizes the need for the complete removal of hydrogen from the silver.
